# Machine learning‐based radiomics in neurodegenerative and cerebrovascular disease

**DOI:** 10.1002/mco2.778

**Published:** 2024-10-28

**Authors:** Ming‐Ge Shi, Xin‐Meng Feng, Hao‐Yang Zhi, Lei Hou, Dong‐Fu Feng

**Affiliations:** ^1^ Department of Neurosurgery Shanghai Jiao Tong University Affiliated Sixth People's Hospital South Campus Shanghai China; ^2^ International Medical College of Chongqing Medical University Chongqing China; ^3^ Anhui University of Science and Technology School of Medicine Huainan China

**Keywords:** machine learning, neuroimaging, poststroke cognitive impairment, radiomics, stroke

## Abstract

Cognitive impairments, which can be caused by neurodegenerative and cerebrovascular disease, represent a growing global health crisis with far‐reaching implications for individuals, families, healthcare systems, and economies worldwide. Notably, neurodegenerative‐induced cognitive impairment often presents a different pattern and severity compared to cerebrovascular‐induced cognitive impairment. With the development of computational technology, machine learning techniques have developed rapidly, which offers a powerful tool in radiomic analysis, allowing a more comprehensive model that can handle high‐dimensional, multivariate data compared to the traditional approach. Such models allow the prediction of the disease development, as well as accurately classify disease from overlapping symptoms, therefore facilitating clinical decision making. This review will focus on the application of machine learning‐based radiomics on cognitive impairment caused by neurogenerative and cerebrovascular disease. Within the neurodegenerative category, this review primarily focuses on Alzheimer's disease, while also covering other conditions such as Parkinson's disease, Lewy body dementia, and Huntington's disease. In the cerebrovascular category, we concentrate on poststroke cognitive impairment, including ischemic and hemorrhagic stroke, with additional attention given to small vessel disease and moyamoya disease. We also review the specific challenges and limitations when applying machine learning radiomics, and provide our suggestion to overcome those limitations towards the end, and discuss what could be done for future clinical use.

## Graphical abstract

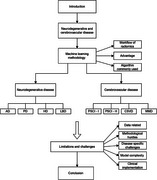

## 1 INTRODUCTION

Cognitive impairments represent a growing global health crisis with far‐reaching implications for individuals, families, healthcare systems, and economies worldwide. As a leading cause of reduced quality of life and lost productivity, their disease burden is substantial and rapidly increasing.^1^, ^2^, ^3^, ^4^ In 2022, the global cost of cognitive impairment was estimated at $1.3 trillion, projected to surpass $2.8 trillion by 2030.^5^ In the United States alone, the annual cost of Alzheimer's disease (AD) and other dementias is expected to reach $1.1 trillion by 2050, potentially overwhelming the healthcare system and impacting economic growth.^6^ The cost of cognitive impairment following cerebrovascular disease is also tremendous. For example, the economic cost of poststroke care and cognitive rehabilitation ranging from $4850 per month in the United States to $752 per month in Australia.^7^ In China, stroke survivors face catastrophic incremental healthcare expenditures, accounting for 57.4% of the unemployment rate.^8^ These conditions profoundly affect the quality of life, independence, and dignity of millions, while placing immense strain on families and caregivers. The progressive nature of many cognitive impairments underscores the crucial importance of early diagnosis and intervention for managing symptoms and potentially slowing disease progression.

Given these challenges, there is an urgent need for more effective methods of early detection, diagnosis, and prognosis in cognitive impairments. The detection and diagnosis model focus on classifying the disease from other conditions with overlapping presentation. While the prognosis model focuses on prediction of the likely course and outcome of a disease or condition based on the diagnosis, including the recovery trajectory, treatment sensitivity, and long‐term management. While previous research has focused on biochemical, genetic, neuroimaging, and neurophysiological predictors, the traditional linear and unidimensional analysis approach are limited in handling multidimensional and non‐linear data, restricting their generalizability, accuracy, and real‐life clinical application.^9^, ^10^, ^11^

Machine learning (ML) has emerged as a powerful tool to address these limitations. By leveraging large datasets and complex algorithms, it can uncover subtle patterns and relationships that may not be apparent through traditional analysis methods.^12^ The advancement of computational power further enhances ML's ability to reveal important features from complex, high‐dimensional data across multiple domains. This could lead to earlier and more accurate diagnoses, personalized treatment strategies, and a deeper understanding of the underlying mechanisms of cognitive impairments and dementia.^11^, ^13^, ^14^, ^15^ To date, the application of ML in cognitive impairments and dementia can be broadly categorized as following: (1) biomarker discovery and validation; (2) diagnosis and detection facilitating clinical decision making and prevention; and (3) prediction of prognosis and disease progression. Those research effectively bridges the gap between complex neurological data and actionable clinical insights, potentially revolutionizing the field of cognitive health and dementia care.

Despite the abundance of studies utilizing ML in cognitive impairments, a comprehensive review that systematically organizes these applications based on the underlying disease types is lacking. Based on the latest review,^16^ cognitive impairment can be broadly caused by neurodegenerative disease and vascular disease. Therefore, our approach divides the field into two main categories according to the etiology of cognitive impairment: neurodegenerative disease and cerebrovascular diseases. Within the neurodegenerative category, this review primarily focuses on AD, while also covering other conditions such as Parkinson's disease (PD), Huntington's disease (HD), and Lewy body dementia (LBD). In the cerebrovascular category, we concentrate on poststroke cognitive impairment (PSCI), including ischemic and hemorrhagic stroke, with additional attention given to cerebral small vessel disease (CSVD) and moyamoya disease (MMD). Through this disease‐based organization, we intend to offer valuable insights into the specific applications and potential of ML across various etiologies of cognitive impairment and dementia.

## 2 NEURODEGENERATIVE AND CEREBROVASCULAR DISEASE

Cognitive impairment can result from various underlying causes, reflecting the complexity of brain function and its susceptibility to different pathological processes. Two main areas of research focusing on radiomic analysis are neurodegenerative diseases and cerebrovascular diseases, both of which can lead to significant cognitive decline but through distinct mechanisms.

### 2.1 Neurodegenerative disease

Neurodegenerative diseases are a group of disorders characterized by progressive degeneration of neurons in specific brain regions.^17^, ^18^, ^19^, ^20^ These diseases, including AD, PD, HD, and LBD, significantly impact older populations. Approximately 54% of elderly patients presenting with cognitive symptoms are diagnosed with a neurodegenerative disorder.^21^

The hallmark of neurodegenerative diseases is the accumulation of abnormal protein aggregates in the brain. In AD, this manifests as amyloid plaques and neurofibrillary tangles, while α‐synuclein aggregates are characteristic of PD and LBD. These protein accumulations lead to synaptic dysfunction, neuronal loss, and ultimately, cognitive decline.

Cognitive impairment in neurodegenerative diseases typically follows a pattern that mirrors the trajectory of brain atrophy associated with disease progression. In AD, for instance, early memory impairment often correlates with initial atrophy in the hippocampus and entorhinal cortex.^22^ As the disease advances, other cognitive domains become affected, reflecting the spread of pathology to other brain regions. The progression of cognitive decline can be categorized into stages, beginning with a preclinical stage characterized by subtle cognitive changes often undetectable through standard cognitive tests. This is followed by mild cognitive impairment (MCI), where noticeable cognitive decline occurs but does not significantly interfere with daily activities. The final stage is dementia, marked by severe cognitive impairment that significantly impacts daily functioning.^23^

The rate of progression varies among individuals and diseases. For example, the median survival time from onset of dementia in AD is 3.3 years for men and 3.7 years for women.^22^ This variability in disease course presents challenges for prognosis and treatment planning, necessitating individualized approaches to patient care.

### 2.2 Cerebrovascular disease

Cerebrovascular diseases, on the other hand, encompass a range of conditions that affect the blood vessels in the brain, accounting for 15%−30% of cognitive impairment cases in populations over 65 years of age.^24^ These conditions, including stroke, CSVD, and MMD, cause cognitive impairment through disruption of blood flow to the brain^17^, ^25^, ^26^

Stroke, a major cause of cerebrovascular cognitive impairment, affects approximately 13.7 million people worldwide each year.^24^ PSCI is particularly prevalent, affecting more than 70% of stroke survivors. The cognitive effects of stroke can be profound even with relatively small lesions if they occur in strategic areas critical for cognitive function, such as the thalamus or hippocampus.^27^, ^28^

The cognitive profile in cerebrovascular diseases can vary depending on the location and extent of vascular damage. Strategic infarct dementia results from small infarcts in critical brain regions, while multi‐infarct dementia occurs due to multiple infarcts throughout the brain. Subcortical vascular dementia primarily affects subcortical structures, often resulting in executive dysfunction and slowed processing speed.

Unlike the gradual progression seen in most neurodegenerative diseases, cognitive changes in cerebrovascular diseases can occur suddenly^29^, ^30^ (as in stroke) or develop insidiously over time (as in CSVD).^31^ The heterogeneity in presentation and progression poses challenges for diagnosis and management, requiring a nuanced approach to patient assessment and care.

## 3 MACHINE LEARNING METHODOLOGY AND APPLICATION IN RADIOMICS

### 3.1 Radiomic workflow

**FIGURE 1:**
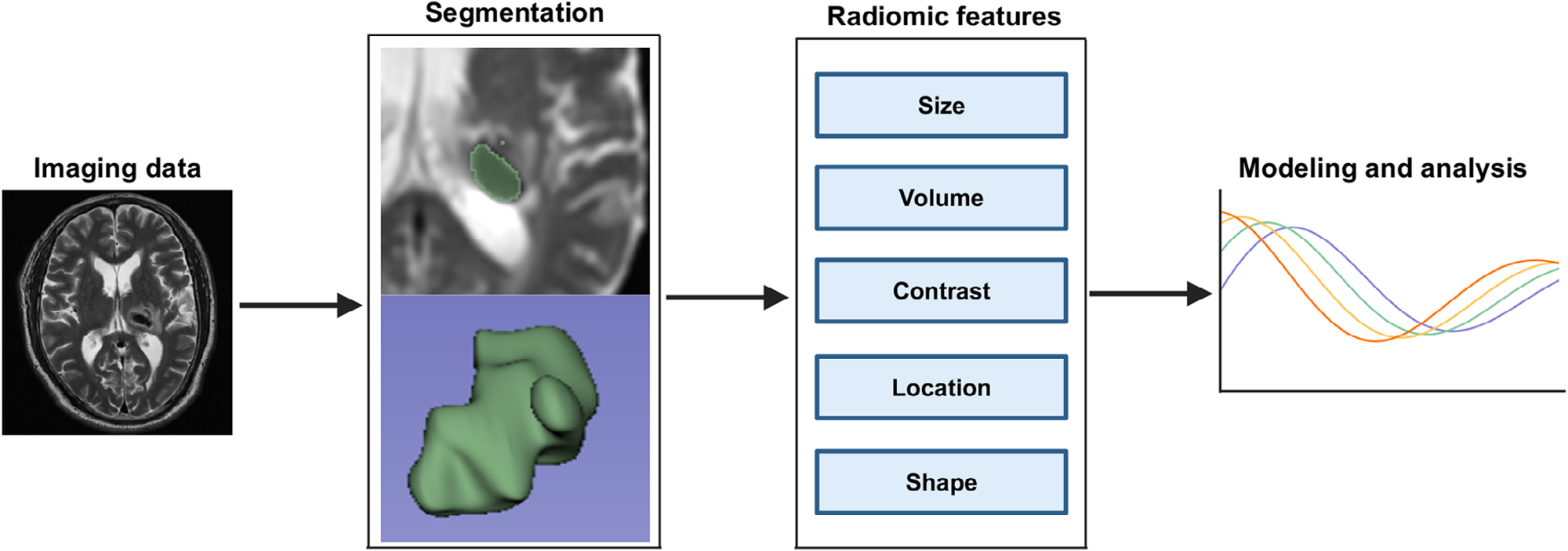
Illustration of the typical workflow in radiomics. *Note*: The example was drawn from a magnetic resonance (MR) image of intracerebral hemorrhage.

Typical radiomic workflow first involves the computerized extraction of innumerable quantitative data from imaging modalities such as computed tomography (CT), magnetic resonance imaging (MRI), or positron emission tomography (PET) into a high‐dimensional, mineable feature space using several data characterization algorithms.^32^ Those critical features can be categorized as semantic and agnostic metrics.^29^ Semantic metrics refer to a set of features that can be captured by the naked eye such as shape or irregularity of the lesion. Agnostic metrics are calculated by mathematical or ML algorithms that constitute the main body of radiomic features. Unlike sematic features, they cannot be obtained through visual interpretation, such as gray‐level frequency from the pixel intensity, interrelationships between pixels, and high‐order statistical descriptors, such as busyness, contract, and coarseness. Then, those extracted data can be used to incorporate other features, such as demographical data or be used alone, to develop predictive or prognostic models to assist clinical decision making, or can be used as an exploratory technique to investigate underlying biomarker for certain disease (Figure 1).

### 3.2 Advantage of using machine learning compared to conventional approach

**FIGURE 2:**
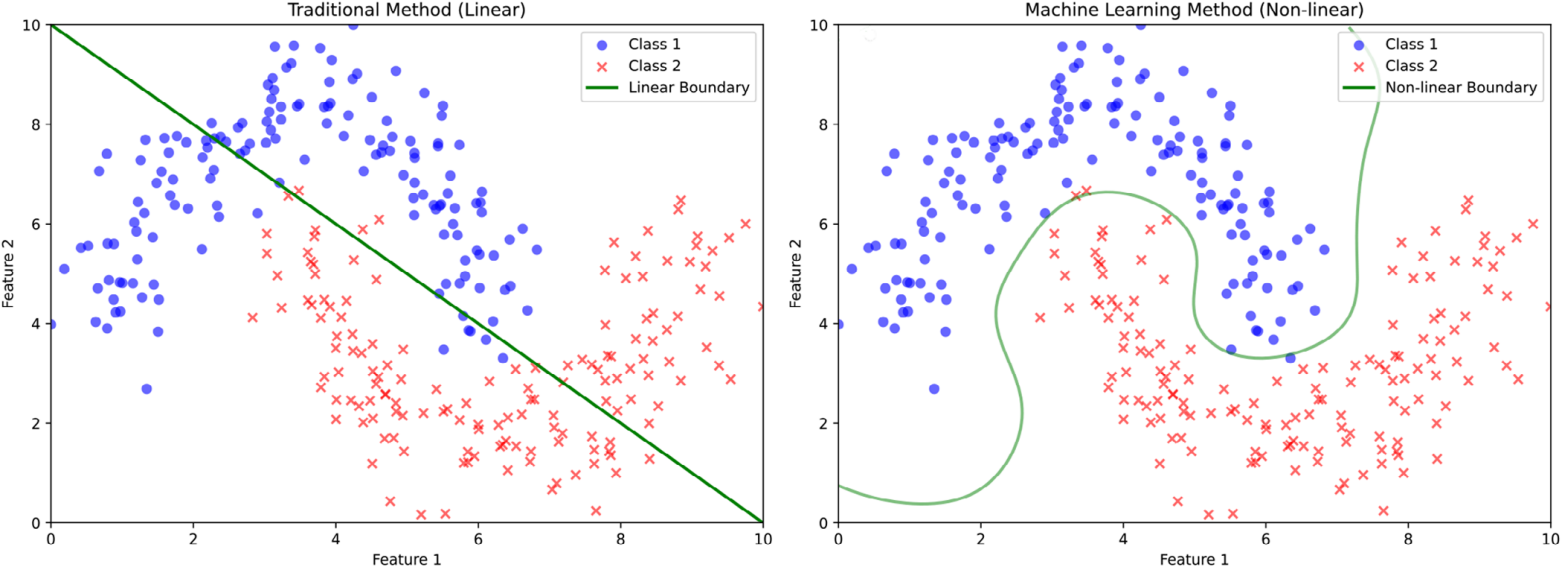
Classification performance of machine learning approach and traditional linear approach. *Note*: The data are artifically generated for illustration purpose only.

ML techniques can be incorporated into the modeling processes. The most pronounced advantage of ML algorithms is evident from their classification performance, which is crucial in applications such as assisting diagnosis or predicting binary clinical outcomes. Figure 2 illustrates the classification performance between traditional linear classification and ML‐based classification. Traditional classification methods, such as logistic regression, assume linear classification boundaries, which cannot capture the complexity of clinical data and its associated cognitive presentation.^34^, ^35^, ^36^, ^37^ For instance, age, a basic demographic feature, is not linearly related to cognitive impairments; the risk may increase exponentially after a certain age threshold.^38^, ^39^ When combined with other clinical features or imaging data, linear classification becomes even less effective in real‐life clinical conditions. In contrast, ML‐based classification can handle non‐linear decision boundaries, capturing more nuanced details in complex data and providing more accurate classification.

ML models also excel at handling high‐dimensional, multivariate data, where the number of features is large relative to the number of samples.^40^, ^41^, ^42^, ^43^ Current research often aims to build comprehensive hybrid models to predict cognitive outcomes based on multiple features including neuroimaging data, genomic data, and laboratory features. Neuroimaging data alone usually involves millions of voxels in an MRI scan. When incorporating other features, classical modeling techniques often result in overfitting and multicollinearity. The univariate nature of traditional analysis further ignoring critical inter‐relationships between predictors. ML models, however, incorporate regularization to prevent overfitting.^44^, ^45^, ^46^ Deep learning techniques, such as convolutional neural networks (CNNs), use weight sharing and pooling to drastically reduce the number of parameters compared to fully connected networks. These techniques enable ML to effectively handle high‐dimensional image data, capturing more inter‐relationships between predictors, which translates to better predictive performance and improved generalizability in clinical settings.

Furthermore, ML can capture patterns of features not typically visible to the naked eye. This is particularly important in neuroimaging analysis, where certain features such as gray‐level frequency from pixel intensity, interrelationships between pixels, and high‐order statistical descriptors such as busyness, contrast, and coarseness are impossible to inspect visually. Deep learning techniques, such as CNNs, can directly extract features from raw imaging input and use computer vision to transform imaging modalities such as CT, MRI, or PET into a high‐dimensional, mineable feature space using various data characterization algorithms.^47^, ^48^, ^49^ These extracted features are then weighted through the algorithm for classification and prediction, resulting in more accurate and objective feature selection, further benefiting model building and predictive power.

### 3.3 Common machine learning algorithm used in the field

**FIGURE 3:**
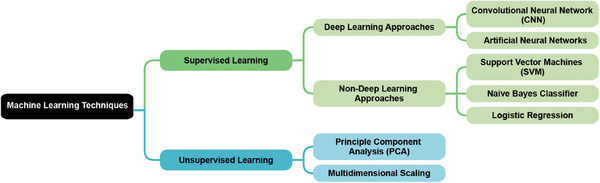
Common machine learning techniques used in radiomic research.

The application of ML techniques in cognitive impairment is primarily based on supervised learning. Models are trained on datasets pairing input clinical data (such as MRI scans, blood test results, or other medical measurements) with known cognitive impairment outcomes. These models learn to identify patterns and relationships between the input data and cognitive status, aiming to accurately predict the likelihood or presence of cognitive impairment in new, unseen patients.^50^ Unsupervised learning is often applied as a preprocessing step to reduce the dimensionality of high‐dimensional clinical data without using cognitive impairment labels.^51^, ^52^ This approach preserves important information while mitigating the curse of dimensionality, improving computational efficiency, and potentially enhancing the performance of subsequent supervised learning models. Deep learning, a subset of ML that can be applied to supervised and unsupervised learning, uses artificial neural networks with multiple layers to automatically learn complex patterns from large‐scale clinical data.^53^ These networks can process diverse inputs such as neuroimaging data, genetic information, and various biomarkers to identify subtle features and non‐linear relationships predictive of cognitive decline. Figure 3 demonstrates the family of common ML techniques used in radiomic research.

## 4 APPLICATION OF MACHINE LEARNING IN COGNITIVE IMPAIRMENT UNDER NEURODEGENERATIVE DISEASE

### 4.1 Alzheimer's disease

**FIGURE 4:**
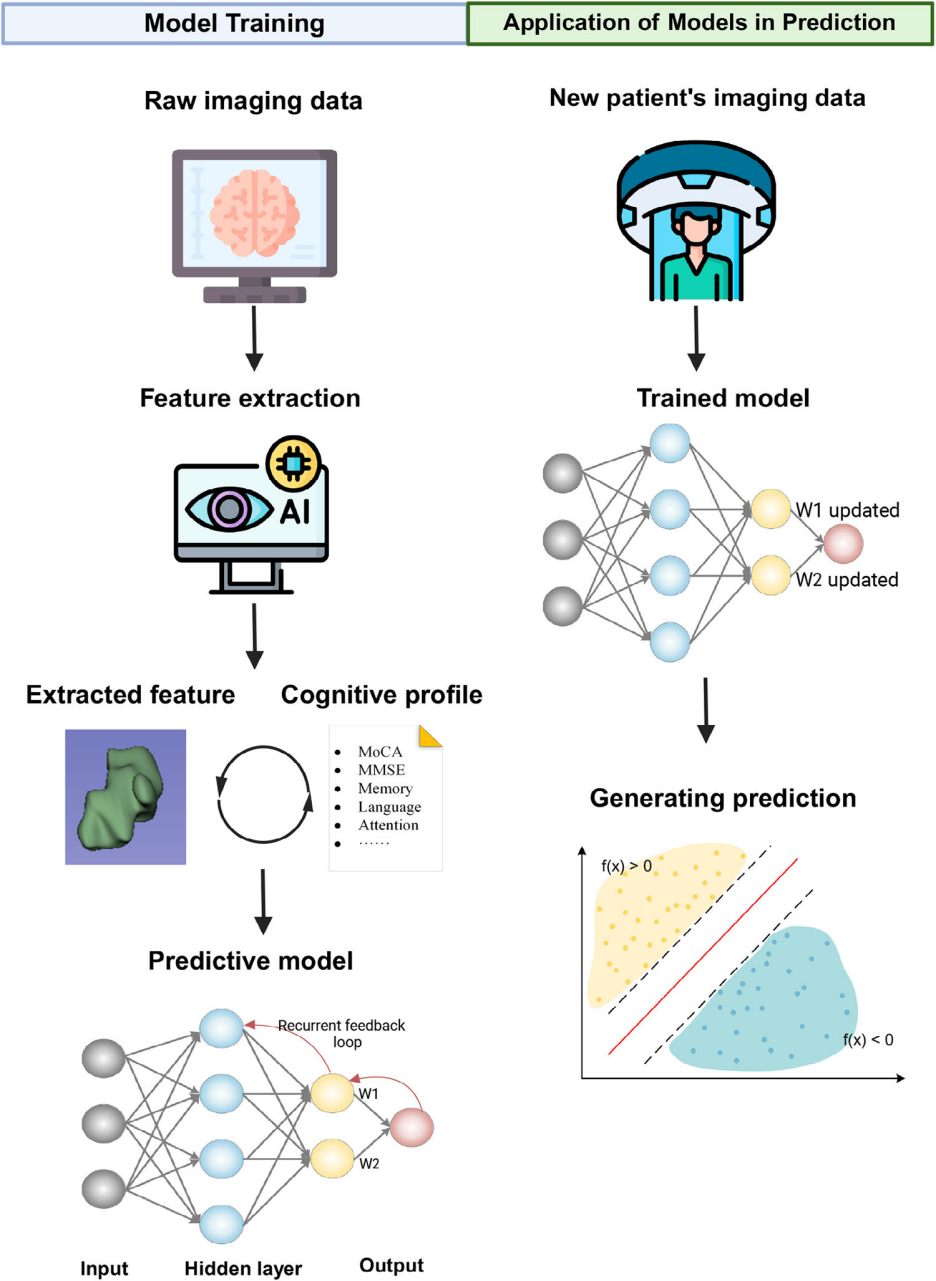
Simplified illustration of predictive modeling based on machine learning radiomics.

AD is a neurodegenerative disorder characterized by two primary pathological hallmarks: the accumulation of amyloid‐β (Aβ) plaques and the formation of neurofibrillary tangles composed of hyperphosphorylated tau protein.^54^, ^55^, ^56^ These pathological changes can be detected in vivo through various neuroimaging techniques, including amyloid and tau PET. Additionally, structural MRI can reveal generalized and focal atrophy, as well as white matter lesions associated with Aβ and tau accumulation. These neuroimaging features form the foundation for radiomic analysis of cognitive impairment in AD (Figure 4).

**FIGURE 5:**
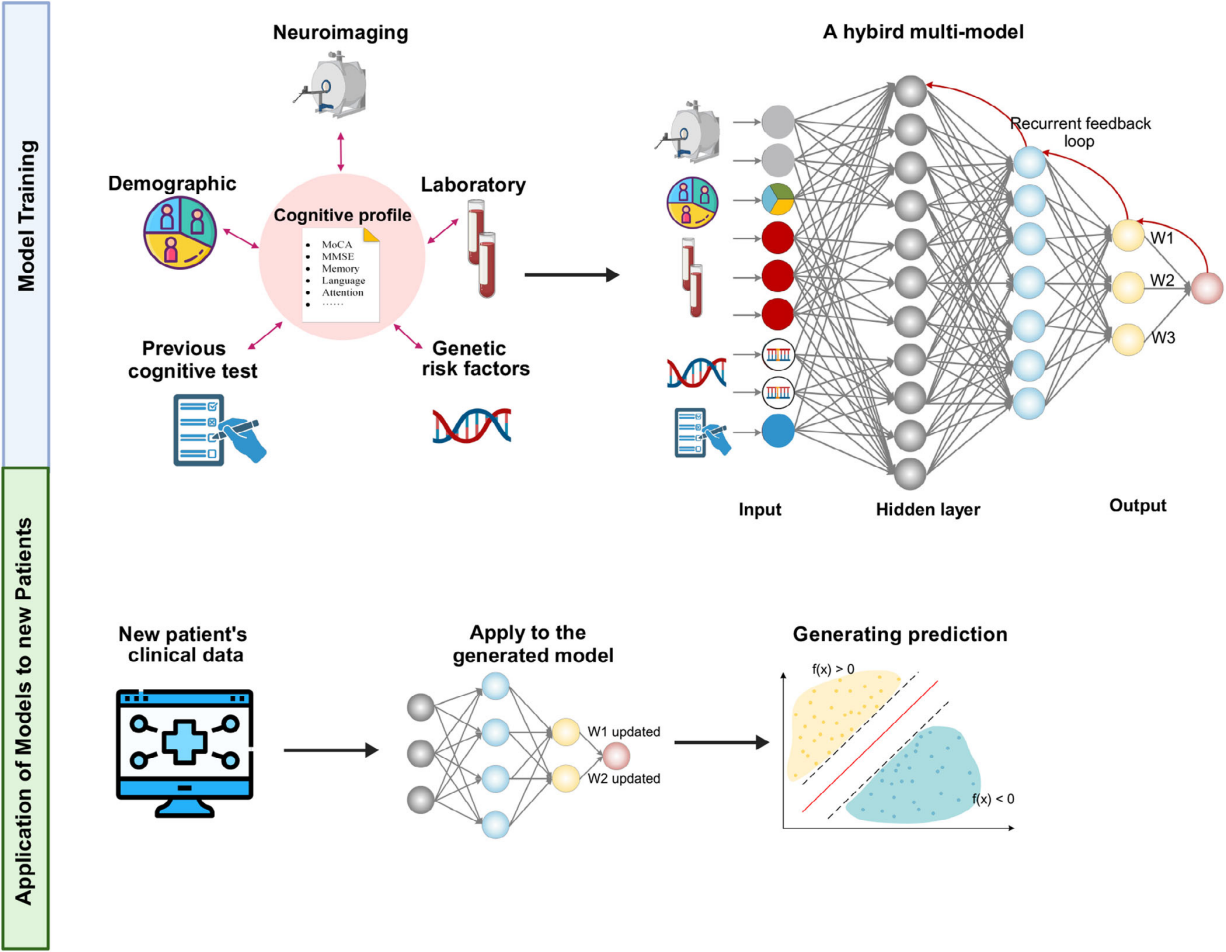
Simplified illustration of a hybrid multimodel for predicting cognitive outcomes.

Beyond neuroimaging, laboratory tests such as cerebrospinal fluid (CSF) analysis (e.g., reduced CSF Aβ) and blood biomarkers (e.g., neurofilament light) provide additional positive biomarkers for AD. Current predictive models also incorporate demographic features, and in some cases, genetic testing results, to create comprehensive prediction models (Figure 5).

#### 4.1.1 PET‐based radiomic approach

There has been a growing interest in using the cerebral glucose metabolism rate for AD classification and prediction of MCI and AD (Table 1). Therefore, many researchers use PET‐based radiomic features to predict and classify AD. For example, a study^57^ utilized support vector machines (SVM) to analyze Fluorodeoxyglucose (FDG)‐PET data aiming to classify AD versus normal subjects. They also compared the model's performance with general linear model and scaled sub‐profile modeling. They identified the critical distribution of hypometabolism for classification in precuneus, medial frontal lobes, the temporal lobes, and the cingulum (posterior and anterior). Their results suggest that ML modeling has the best predictive performance with 0.84 in sensitivity and 0.95 in specificity. Furthermore, their results suggest that the classification could be further generalized to other types of neurodegenerative diseases such as PD and LBD, which may indicate the importance of a shared cholinergic system deficit.

**TABLE 1:** Example of literature using positron emission tomography (PET)‐based radiomic features in Alzheimer's disease (AD).

Literature	Modality	Classification	Algorithm	Accuracy
^53^	Tau‐PET	AD/CN	3D‐CNN	90.80%
^58^	FDG‐PET	AD/CN	Multi‐kernel probability classifier	94.03%
^59^	Amyloid‐PET	AD/CN	2.5D‐CNN	75.00%
^60^	FDG‐PET	AD/MCI	2D‐CNN	51.80%
^61^	FDG‐PET	AD stage	SVM	96%
^62^	FDG‐PET	AD/MCI/CN	CNN	75%
^63^	PiB‐PET + FDG‐PET	AD/CN	SVM	82.71%
^64^	High‐dimensional PET	AD/CN	Random forest + SVM	95.45%

Abbreviations: CNN, convolutional neural network; MCI, mild cognitive impairment; SVM, support vector machine.

Beyond classifying AD from healthy controls, recent studies have also focused on classifying AD from other neurodegenerative diseases. For example, Nancy Noella and Priyadarshini applied naïve Bayes, decision tree, SVM, and bagged ensemble classifier aiming to classify AD from PD based on FDG‐PET data.^65^ Among these, the bagged ensemble classifier yields an accuracy of 90.3%, and the sensitivity, specificity, and precision values of the classifier are 0.89, 0.92, and 0.87 respectively. Similarly, Tufail et al. applied a 3D‐CNN architecture based on PET and single‐photon emission computed tomography (SPECT) aiming to classify AD, PD, and normal control.^62^ Their model achieved a 95.97% index of balanced accuracy, which further highlights the ability of ML in automatically processing raw imaging data.

Additional work has been done to predict the conversion from MCI to AD. For example, Zhou et al.^66^ applied SVM classifier aiming to predict potential conversion to AD based on FDG‐PET.^62^ Their results indicate a 90% accuracy with 87.5% sensitivity and 93.39% specificity, which suggests the feasibility to predict conversion to AD in MCI patients.

While FDG‐PET focuses on metabolism, amyloid PET provides direct visualization of amyloid plaques which contribute to the development of the disease. For example, Segovia et al. construct an support vector regression (SVR) model based on the fusion of F‐Florbetaben PET and FDG‐PET aiming to assist the diagnosis of AD.^67^ They found that the combined approach achieved a good trade‐off between sensitivity and specificity and higher accuracy rates than systems based on single‐modality approaches. Furthermore, Nai et al. conduct model comparison between 68 ML methods based on amyloid PET images.^68^ Among all, naïve Bayes classifier performed the best with better convergence between training and unseen data. Their results also suggest that deep learning networks performed poorly for equivocal cases. They further suggest the importance of gray‐scale in detecting focal accumulation, which provides important guidance for further study.

Tau PET can also provide a direct observation regarding the pathology accumulation. For example, Park et al.^69^ developed a 3D‐CNN deep learning network aiming to classify AD, MCI, and healthy control based on tau PET image. Their model achieves area under the curve (AUC) generally greater than 0.90, which shows ML's promising utility in classifying AD stages.^70^ Furthermore, studies such as Gebre et al. introducing an advanced tau summary measure aim to quantify the heterogeneous burden of tau deposition into a single number based on tau PET that would be clinically useful.^71^ They calculate the tau heterogeneity evaluation in AD score using standard uptake value (SUV) ratios and Shapley additive explanations for each participant. Their model achieved a balanced accuracy of 95% on training set and 87% on validation set, highlighting a great potential in clinical use for an accurate identification of tau deposition with easy interpretation.

#### 4.1.2 MRI‐based radiomic approaches

MRI data are also commonly used in radiomic analysis given its ability to manifest the brain atrophy caused by AD (Table 2). Current emphasis was placed on disease identification, such as distinguishing AD from healthy population, given AD's confusability with normal aging. For example, Battineni et al. applied naïve Bayes, artificial neural networks, K‐nearest neighbor (KNN), and SVM on structural MRI data to identify AD from healthy control in older subjects.^72^ The model with best performance is the naïve Bayes classifier with automatic feature selection, which achieved the highest receiver operating characteristic (ROC) of 0.942. Likewise, Saeed and coworkers applied CNN‐based feature selection to build ML models based on SVM and random forest algorithm.^73^ The accuracy is substantially improved, ranging from 85.7% to 99% for all models. The application of machine learning‐based radiomics is therefore promising in identifying the disease from other conditions with similar presentation.

**TABLE 2:** Example of literature using magnetic resonance imaging (MRI)‐based radiomic features in Alzheimer's disease (AD).

Literature	Modality	Classification	Algorithm	Accuracy
^74^	sMRI	AD/CN	SVM	97%
^75^	fMRI	AD/CN	3D‐CNN	85.27%
^72^	fMRI	AD/CN	FNN	81%
^76^	fMRI	MCI/AD	GCN	78.50%
^77^	fMRI + age + APOE4	CN/MCI	Autoencoder	86%
^75^	DTI	AD/CN	SVM + XGBoost	82.35%
^78^	DTI + sMRI	AD/MCI	Naïve Bayes	96.92%
^79^	DTI + sMRI	MCI/AD/CN	ResNet‐10	95.24%/93%
^80^	sMRI	MCI/AD/CN	SVM	94%
^81^	sMRI	MCI/AD	CNN	99.68%

Abbreviations: APOE, Apolipoprotein E; CN, cognitive normal; CNN, convolutional neural network; DTI, diffusion tensor imaging; fMRI, functional MRI; FNN, feed‐forward neural network; GCN, graph convolutional network; MCI, mild cognitive impairment; sMRI, structural MRI; SVM, support vector machine.

Some studies also take a step further from merely disease classification to identify the critical brain areas devoting to the development of the disease. For example, Li et al. applied a 3D‐ResNet approach to identify the critical features and brain areas contributing to the development of AD.^82^ They discovered that anatomic properties, such as volume of cerebral cortex, gray matter density, and the thickness of the cerebral cortex are revealed to be critical for identification. In addition, they found that the cerebellum was highlighted in the maps generated based on deep learning models. However, cerebellum is a relatively neglected area of the AD brain and is not commonly considered involved in the pathophysiology of AD. Therefore, their results may suggest that cerebellum has roles in the clinical phenomenology of the disease.

Other than structural lesions, functional abnormality of the brain in AD patients is also commonly observed under functional MRI (fMRI) imaging. For example, Ramzan et al. applied a deep learning approach to resting‐state fMRI (rs‐fMRI) data aiming to classify all stages of AD progression. Their models achieve accuracy of 100%, 96.85%, 97.38%, 97.43%, 97.40%, and 98.01% for each stage of AD respectively.^83^ Similarly, Alorf and Khan applied a deep learning method further revealing the critical brain regions with abnormal functionality contributing to the presentation via extraction weight.^84^ Their models achieve an accuracy of 92% and indicate the important features including middle frontal gyrus, superior frontal gyrus, precentral gyrus, superior frontal gyrus, and inferior frontal gyrus for the classification.

Zhu et al. further examine the hippocampal functional connectivity, which is known to contribute significantly to the cognitive impairment, especially in memory domine, in different stages of AD based on SVM models.^85^ Their models achieve an overall accuracy higher than 80% and indicate that the hippocampus and left insula, left thalamus, cerebellum, right lingual gyrus, posterior cingulate cortex, and precuneus were significantly reduced in AD and MCI. fMRI data can also provide evidence to the causal relationship between brain atrophy and disease progression. More recent studies also aim to build causal modeling based on fMRI data through ML techniques. For example, Wang applied a novel ML‐based causality method, namely, the new causality with fMRI data, aiming to reveal critical brain areas that cause the impairment in AD patients.^86^ They identify middle insular cortex, inferior parietal cortex, and lateral temporal cortex as key roles in consciousness and functionality diversion.

#### 4.1.3 Combined neuroimaging approaches

Some research also combines MRI and PET as training data for a more comprehensive neuroimaging analysis. For example, Zhao et al. used a deep learning approach analyzing the combination of PET and MR data to provide both functional and structural information of the brain.^87^ The proposed model reaches an accuracy of 95.65%, which exhibited clinical validity and possessed the potential for application. Likewise, Subramanyam Rallabandi and Seetharaman compared multiple deep learning models' performance using MRI/PET combined data and suggest that ResNet‐50 model's performance surpasses any existing algorithm in current literature when handling such data within AD condition, with an accuracy generally above 95%.^88^

Furthermore, some studies integrate several ML algorithms (e.g., SVM + RF + Ada Boost + KNN) for modeling using MRI/PET combined data.^89^, ^90^, ^91^ Their model all results in an impressive accuracy of 95% in their dataset. In their following work, they used similar methods based on combined neuroimaging data to reveal that right hemisphere's parahippocampal and entorhinal regions were the most influential features contributing to the detection of AD, MCI, and healthy control.^92^

#### 4.1.4 Hybrid‐multimodal approaches

In more recent studies, integrating neuroimaging data with clinical data, such as CSF biomarkers, blood tests, and cognitive assessments, has become a promising approach in the diagnosis of AD.^93^, ^94^, ^95^, ^96^, ^97^ These multimodal approaches leverage ML algorithms to process and analyze diverse datasets, leading to improved accuracy in predicting disease progression, identifying at‐risk individuals, and potentially guiding therapeutic interventions.

For example, Kumar et al. utilized an SVM classifier to combine MRI neuroimaging data with clinical measures, including cognitive test scores, CSF biomarkers, and blood‐based biomarkers.^98^ Their model achieved an accuracy of 87%, with an area under the ROC curve of 0.89. The study identified key features contributing to the model's predictive power, such as hippocampal volume (from MRI) and levels of CSF tau and Aβ. These features are critical as they are directly linked to the pathophysiological processes of AD. Similarly, Shmulev and Belyaev use XGBoost algorithm to predict the conversion of MCI to AD.^99^ The model integrated neuroimaging data, including cortical thickness and hippocampal atrophy, with clinical features such as age, genetic markers (APOE ε4 status), and cognitive scores. The model achieved a prediction accuracy of 76%, with an ROC AUC of 0.86. Important features identified in the study were cortical thickness in specific brain regions and APOE ε4 status, both of which are known risk factors for AD progression.

While the CSF sample provides valuable information regarding the AD pathology, it may be limited for its cost and complex examining procedure. More accessible features, such as blood plasma, could be investigated for its predictive value for AD. For example, Karlsson et al. developed a CNN model to predict tau load and distribution in AD by combining plasma biomarkers (such as p‐tau and neurofilament light chain), structural MRI data, and clinical variables.^100^ The model achieved an accuracy exceeding 90% across multiple test cohorts. The study highlighted the importance of integrating non‐invasive plasma biomarkers with MRI features such as gray matter volume and white matter hyperintensities, which were key predictors in the model. These features are crucial as they provide insights into the neurodegenerative processes at play in AD.

The implications of these studies are profound, as they demonstrate that integrating neuroimaging with clinical data significantly improves the predictive accuracy of ML models. This approach not only enhances the early detection of AD but also offers insights into the underlying mechanisms of disease progression, paving the way for more personalized treatment strategies. The identification of key features such as hippocampal volume, CSF biomarkers, and genetic markers further underscores the importance of these multimodal approaches in understanding and managing AD.

### 4.2 Parkinson's disease

PD is also a progressive neurodegenerative disorder primarily characterized by the loss of dopaminergic neurons in the substantia nigra pars compacta. This neuronal loss leads to the classic motor symptoms of PD, including tremor, rigidity, bradykinesia, and postural instability. However, PD also presents with various non‐motor symptoms, including cognitive impairment, which can significantly impact patients' quality of life. The pathological hallmark of PD is the presence of α‐synuclein‐containing Lewy bodies in surviving neurons.

Recent advancements in neuroimaging, combined with the application of ML techniques, have enabled more precise and earlier diagnosis of PD, as well as improved monitoring of disease progression. Structural MRI, fMRI, and dopamine transporter (DAT) imaging using SPECT or PET, provide valuable insights into the neurodegenerative processes in PD. These neuroimaging modalities form the basis for radiomic analyses that leverage ML algorithms to detect subtle changes in brain structure and function that are characteristic of PD.

#### 4.2.1 Dopamine transporter imaging and PET‐based approaches

DAT imaging, particularly utilizing PET, has emerged as a powerful tool in the study of PD. ML and radiomic approaches are increasingly applied to these imaging techniques, enhancing diagnostic accuracy and providing deeper insights into disease progression and differentiation. A notable example is the application of deep CNNs in the differential diagnosis of parkinsonism using DAT imaging. In a study by Zhao et al., a CNN model demonstrated an impressive area under the receiver operating characteristic curve (AUROC) of 0.953, with a sensitivity of 87.7% and a specificity of 93.2% during cross‐validation.^101^ These results underscore the potential of ML models to accurately distinguish between different types of parkinsonism, which is critical given the overlapping clinical presentations of these disorders.

Different ML algorithms have also been tested to investigate their feasibility and accuracy to detect PD from normal controls. For example, Wu et al. employed radiomic features combined with SVM to differentiate PD patients from normal controls using 18F‐FDG‐PET imaging.^102^ The model achieved accuracies of 90.97% and 88.08% across different test sets. This high level of accuracy further supports the utility of radiomics and ML in refining neurodegenerative disease diagnostics. Nakajima et al. explored the use of various ML algorithms, including logistic regression, KNN, and gradient boosted trees, to differentiate abnormal profiles on 123I‐ioflupane images.^103^ These models all achieved high accuracy (AUC 0.92‒0.96) and were able to accurately diagnose Parkinson's syndrome, PD, and dementia with Lewy bodies (DLB), further validating the role of ML in enhancing the diagnostic capabilities of DAT imaging. They also identified the KNNs’ algorithm as the most accurate model in this context, highlighting its potential for clinical use.

Further innovation has also been proposed to enable a more efficient model training process. For example, Shao et al. utilized a generative adversarial network (GAN) to create digital brain phantoms for training ML algorithms in PD SPECT imaging.^104^ This approach addresses the challenge of needing large amounts of data for training ML models and demonstrates innovative ways in which ML can be applied to neurodegenerative disease research

#### 4.2.2 Structural MRI‐based approach

Structural MRI‐based radiomics, when combined with ML techniques, provides a powerful tool for understanding the cognitive decline associated with PD. The ability to extract and analyze quantitative imaging features from structural MRI scans offers insights into subtle brain changes that correlate with cognitive dysfunction in PD patients, often undetectable through conventional imaging methods.

There are several studies that aim for early detection of PD. For example, A key study by Luo et al. explored the predictive capability of radiomic features derived from T1‐weighted MRI in identifying early signs of levodopa‐induced dyskinesia, a condition closely linked with cognitive decline in PD.^105^ By integrating these radiomic features with clinical data, the study achieved a high accuracy of 87.5% in its predictive model. This suggests that early structural changes in the brain might be linked to both motor and cognitive dysfunctions, allowing for earlier intervention and better management of cognitive symptoms in PD. Furthermore, Sun et al. combined MRI radiomics with PET imaging to distinguish PD from multiple system atrophy (MSA).^106^ Their multimodal approach, integrating structural MRI with PET, achieved an accuracy of 88.6% in differentiation. The study highlighted how specific structural changes in the brain, particularly in regions associated with cognitive functions, are essential for accurate diagnosis, reflecting the differences in cognitive domains affected by PD and MSA.

Extending from early detection, other studies have tried to identify different subtypes of PD, which would provide a more nuanced diagnosis and enable a more specified treatment. For example, Quattrone et al. utilized ML algorithms such as XGBoost and random forest to differentiate between progressive supranuclear Palsy‒Richardson's syndrome (PSP‐RS) and PSP‐Parkinsonism (PSP‐P).^107^ The models achieved a high AUC of 0.93 ± 0.04, reflecting their effectiveness in distinguishing these subtypes, which are often associated with varying degrees of cognitive impairment. This accuracy underscores the potential for MRI‐based radiomics to serve as biomarkers for cognitive impairment in PD, allowing for more precise differentiation of disease subtypes based on cognitive profiles.

After the diagnosis, the responsiveness of treatment is also critical for personalized care. Haliasos et al. leveraged whole‐brain white matter radiomics to personalize deep brain stimulation (DBS) therapy for PD patients.^108^ Their random forest model demonstrated a prediction accuracy of 85.2% in optimizing DBS outcomes, with particular relevance to cognitive decline. This finding suggests that radiomics could be instrumental in tailoring therapeutic interventions aimed at mitigating cognitive deterioration in PD, thus enhancing patients' quality of life.

#### 4.2.3 Functional MRI‐based approach

Functional MRI‐based radiomics has emerged as a critical approach in the study of PD, offering valuable insights into the functional alterations in the brain that underlie motor and cognitive symptoms. By leveraging ML techniques, radiomics allows for the extraction of complex patterns from functional MRI data, providing a deeper understanding of disease mechanisms and aiding in the development of predictive models.

Effort has been made to use the functional imaging for disease classification, which aids the clinical diagnosis. For example, A study by Shi et al. utilized ML radiomics to classify PD patients and healthy controls using amplitude of low‐frequency fluctuation‐based features derived from rs‐fMRI.^109^ The study achieved an accuracy of 81.45% and an AUC of 0.850 in the primary dataset, with good generalization in the external validation set (accuracy of 67.44%, AUC of 0.667). These results demonstrate the potential of functional MRI radiomics in distinguishing between PD patients and healthy individuals, highlighting its value in early diagnosis and disease monitoring.

Another innovative approach by Mellema and Montillo^110^ introduced an ML‐based measure of functional connectivity using fMRI, which attained high reproducibility with a mean intra‐subject *R* ^2^ of 0.44. This technique enhances the reliability of fMRI data analysis, making it a robust tool for assessing functional brain networks associated with cognitive decline in PD.

fMRI data are also used for predicting the treatment responsiveness after the diagnosis, which is critical for personalized care. For example, Boutet et al. applied ML to functional MRI data to predict optimal DBS parameters for PD patients, achieving an accuracy of 88%.^111^ This study underscores the utility of functional MRI radiomics not only in understanding disease progression but also in personalizing treatment strategies. By predicting DBS parameters that can improve both motor and cognitive outcomes, this approach offers a way to enhance patient care.

#### 4.2.4 Hybrid‐multimodal approaches

Hybrid ML models that integrate neuroimaging data with various clinical features, such as CSF biomarkers, genetic profiles, and blood test results, have shown significant potential in advancing the diagnosis, prognosis, and treatment of PD. These multimodal approaches leverage the strengths of each data type, leading to more accurate and comprehensive assessments.

A study by Almgren et al. developed a multimodal model based on SVM to predict continuous cognitive decline in early PD.^70^ This model incorporated multiple clinical test scores, such as the Unified Parkinson's Disease Rating Scale (UPDRS) and Mini‐Mental State Examination (MMSE), alongside CSF biomarkers, brain volumes, and genetic variants. Among these, the UPDRS scores were particularly heavily weighted in the model, given their direct correlation with motor symptoms and overall disease severity. Their model achieved high predictive accuracy, with a strong correlation between predicted and observed scores (*r* = 0.44). The integration of neuroimaging with clinical data allowed for a more nuanced understanding of cognitive deterioration, which is often an early and critical aspect of PD progression.

Some innovation in computational modeling technique has also been made. For example, Ding et al. introduced a novel multimodal co‐attention module, integrating embeddings from separate graph views derived from low‐dimensional representations of images and clinical features.^112^ Additionally, they devised a simplified contrastive loss‐based fusion method to enhance cross‐view fusion learning. They combined neuroimaging data with clinical features to classify PD from healthy controls. With their innovation, the model achieved an accuracy of 91%, demonstrating superior predictive capabilities on non‐image data compared to solely ML‐based methods.

### 4.3 Huntington's disease

Striatal and cerebral cortical atrophy are hallmarks of HD progression. Consequently, structural MRI (sMRI) has been extensively applied to radiomic analysis of HD and associated cognitive decline. HD is a monogenic disorder caused by an expanded CAG trinucleotide repeat in the huntingtin (HTT) gene. Genetic testing, which identifies this expansion, remains the definitive diagnostic tool for HD. The resulting mutant HTT protein accumulates, leading to neuronal dysfunction and death. Importantly, the number of CAG repeats correlates with age of onset and disease severity. Given this genetic etiology, most HD‐related radiomic studies employ a multimodal approach, integrating neuroimaging and genetic data to develop comprehensive models for HD prediction and progression tracking.

#### 4.3.1 Structural MRI‐based radiomics

Recent sMRI‐based radiomics studies in HD have predominantly focused on striatal regions, aligning with the characteristic atrophy pattern observed in the disease. Barrett et al. developed a random forest‐based prediction model using volumetric MRI to differentiate HD stages.^113^ Their model achieved an AUC of 0.83, underscoring the pivotal role of striatal volumes in delineating disease progression.

While striatal area is undoubtable important in HD, current research often put extensive focus on this area but overlooked the importance of the other areas. Kohli et al. expanded on this approach by employing nine distinct ML techniques.^114^ They combined volumetric data from multiple brain regions, including the striatum, to predict various stages of HD progression. Their stacking model achieved 82.0% accuracy, demonstrating that while striatal volume remains crucial, volumetric changes in other regions—such as the caudate, putamen, occipital lobe, cingulate cortex, lateral ventricles, and sensorimotor areas—significantly contribute to classification accuracy. Building on these findings, Haase et al. evaluated deep learning‐based models in HD patients, focusing on proposed areas of brain atrophy.^115^ They demonstrated strong correlations between the degree of atrophy in the caudate nucleus, putamen, and pallidum and clinical presentations of cognitive function. Collectively, these studies establish that sMRI‐derived atrophy volumes can effectively track HD progression, serving as robust predictors in ML‐based radiomics models.

#### 4.3.2 Hybrid‐multimodal approaches

Hybrid‐multimodal approaches integrating neuroimaging with clinical data, including genetic and demographic predictors, are emerging as powerful tools in predicting HD progression. These comprehensive methods offer a nuanced understanding of the disease, enhancing prognostic model accuracy and informing clinical trial designs.

Motor impairment, one of the hallmarks of HD development, could be valuable to considered when building a hybrid multimodal. Mohan et al. applied Bayesian latent‐variable analysis to construct a multimodal model based on neuroimaging data and clinical measures, including motor, cognitive, and functional assessments.^116^ Their study incorporated longitudinal data encompassing 2079 assessment measures from four observational studies, making it the largest of its kind to date. The model achieved high accuracy in deriving disease state characteristics and progression probabilities across nine disease stages.

Other from the behavioral measure of HD development, genetic influence is also critical for the prediction, which is more quantifiable and have direct causal relationship. Ghofrani‐Jahromi et al. utilized a random forest algorithm to integrate brain imaging features with genetic, cognitive, and motor biomarkers.^117^ This model achieved a cross‐validated accuracy of 81%, demonstrating a 24% reduction in mean absolute error compared to traditional methods. This improvement in HD patient stratification could significantly impact clinical trial design and patient management.

Some studies has also developed models with more accessible and routine examination. For example, Simmons et al. developed a comprehensive model combining neuroimaging data (MRI and DTI) with laboratory data (urinary and plasma samples) to predict treatment response in HD.^118^ Notably, this study pioneered the incorporation of urinary samples as predictors in HD radiomics. Their model suggested that urinary p75NTR‐ecd levels can detect therapeutic effects, while MRI and plasma cytokine levels may serve as effective pharmacodynamic biomarkers. They proposed that combinations of these markers would be viable and powerful options for clinical trials.

These multimodal studies demonstrate improved predictive power and reveal important features corresponding to disease progression and therapeutic effects in HD. The integration of diverse data types, including neuroimaging, genetic, and biochemical markers, offers a more comprehensive approach to understanding and managing this complex neurodegenerative disorder

### 4.4 Lewy body dementia

LBD is characterized by the accumulation of α‐synuclein protein aggregates, known as Lewy bodies, within neurons. This accumulation leads to widespread neurodegeneration and diverse clinical manifestations. Common neuroimaging modalities for LBD include MRI and SPECT, particularly DAT scans. These scans are essential for differentiating LBD from other neurodegenerative disorders due to their ability to detect dopaminergic deficits. Additionally, PET using specific tracers enhances the visualization of Lewy body pathology.

Beyond neuroimaging, clinical predictors such as detailed cognitive assessments, motor symptom profiles, and biomarkers such as CSF α‐synuclein levels are crucial for accurate diagnosis and prognosis of LBD. Integrating these multimodal data sources facilitates a comprehensive understanding and effective management of LBD.

#### 4.4.1 Dopamine transporter imaging and PET‐based approaches

DAT imaging, utilizing PET and SPECT, is an invaluable tool in the study and diagnosis of LBD. The integration of radiomics with ML techniques in these imaging modalities has significantly enhanced diagnostic accuracy and provided deeper insights into the neurodegenerative processes underlying LBD.

The primary focus was on the disease classification. Many studies aim to use ML‐based model classifying the LBD from normal control. For example, Nakajima et al. utilized ML algorithms, including logistic regression, KNNs, and gradient boosted trees, to differentiate abnormal profiles on 123I‐ioflupane SPECT images.^103^ This study demonstrated high diagnostic accuracy, particularly for a model that integrated imaging features with patient age, achieving an AUC of 0.93 ± 0.02. This finding underscores the potential of ML in accurately distinguishing LBD from other neurodegenerative conditions such as PD and AD, which often share overlapping clinical symptoms.

The feasibility of different imagining morality was also examined. Chiu et al. explored the use of a ML‐derived visual scale (ML‐VS) for Tc99m TRODAT‐1 imaging, which was validated across multiple centers for differentiating LBD from AD and PD.^119^ The ML‐VS model demonstrated an acceptable correlation with traditional diagnostic approaches. Their work was conducted under real‐world clinical settings, suggesting ML's promising potential in assisting clinical decision making and diagnosis. Similarly, Chen et al. investigated the use of β‐amyloid PET and DAT imaging in MCI with core clinical features of LBD.^120^ The study revealed that most patients exhibited low β‐amyloid deposition and reduced dopaminergic activity, providing further evidence that these imaging biomarkers, when analyzed with ML techniques, can be crucial in the early and accurate diagnosis of LBD.

Extending from classification, other studies have also attempted to understand the neurobiology, especially in terms of the brain metabolism, that contribute to the LBD development. For example, Yoo et al. examined the interrelation between nigrostriatal dopamine depletion, brain metabolism, and cognition in LBD using imaging biomarkers and statistical analyses.^121^ The study's findings suggested that ML‐based radiomics could be instrumental in understanding the complex interactions between these factors, thus aiding in the more accurate diagnosis and management of LBD.

#### 4.4.2 Structural MRI‐based approaches

Similar to other neurodegenerative diseases, sMRI is a powerful modality for detecting atrophy as the disease progresses. Therefore, many studies construct radiomic models based on MRI to predict or classify LBD.

Some studies attempt to differentiate various conditions of cognitive impairment with overlapping symptoms. For example, De Francesco et al. introduced the ML algorithm MUQUBIA, designed to differentiate between various forms of dementia, including LBD, using a combination of sociodemographic, clinical, and MRI variables.^122^ The study demonstrated high overall diagnostic performance with an AUC of 98%. The MUQUBIA algorithm successfully classified LBD and other dementias, highlighting its potential utility in clinical settings, where accurate differential diagnosis is critical for appropriate treatment planning. With the support of the utility of MRI‐based radiomics, Minne et al. explored the use of 3D classical versus GAN‐based augmentation for MRI brain images in a multicenter European study, focusing on LBD and AD.^123^ This study highlighted the potential of ML in improving diagnostic accuracy and robustness across diverse datasets.

Redolfi et al. employed 3D‐CNNs deep learning approaches to differentiate between LBD and other neurodegenerative diseases based on MRI imaging.^124^ Their model achieved high classification accuracy, with scores of 0.92 for cognitively normal, 0.90 for AD, 0.97 for DLB, and 0.99 for PD, demonstrating that MRI‐based radiomics combined with advanced ML techniques can significantly enhance diagnostic precision. Ganjizadeh et al. discussed the broader application of AI‐driven radiomics in neurodegenerative diseases, including LBD.^125^ They emphasized that these AI‐driven approaches revolutionize the field of neurodegenerative disease management, enhance diagnostic accuracy, enable early intervention, and ultimately improve patient outcomes. These studies collectively underscore the importance of structural MRI‐based radiomics in advancing the diagnosis and understanding of LBD.

#### 4.4.3 Hybrid‐multimodal approaches

Hybrid ML models that integrate neuroimaging data with various clinical features, such as cognitive scores, genetic markers, and other clinical assessments, have shown significant potential in advancing the diagnosis and management of LBD.

McCombe et al. applied the repeated incremental pruning to produce error reduction (RIPPER) algorithm, incorporating neuroimaging data including MRI and fMRI with neuropsychological testing data including MMSE, along with blood biomarkers, aiming to classify LBD from AD.^126^ Their model achieved a mean accuracy of 94%, suggesting the clinical relevance of simple and interpretable high‐performing ML algorithms. The study identified a variety of readily available clinical assessments for differential diagnosis of dementia, offering opportunities to incorporate various simple and inexpensive screening tests for LBD and addressing the problem of LBD underdiagnosis.

While valuable and informative, MRI data could be limited due to its accessibility and relatively high economic burden. More accessible and cheap neuroimaging alternatives should also be explored. For example, Suzuki et al. validated a ML algorithm that combined quantitative electroencephalogram (qEEG) data with clinical features, including blood tests and cognitive assessments, to differentiate between LBD and AD.^127^ The model achieved an accuracy of 79.5% and a specificity of 85.7%. Their work suggests that diagnostic imaging modality is not limited to MRI; cheaper alternatives, such as EEG, combined with other features can also achieve promising accuracy levels in a more accessible and affordable manner.

In conclusion, Burgos and Colliot emphasized the importance of integrating multimodal data, including neuroimaging, clinical assessments, blood tests, and genetic data, to improve the precision of diagnosis and prognosis in neurodegenerative diseases such as LBD.^128^ Their work highlights ongoing efforts to combine diverse data sources in ML models, aiming to enhance the accuracy and reliability of clinical predictions.

## 5 APPLICATION OF MACHINE LEARNING IN COGNITIVE IMPAIRMENT UNDER CEREBROVASCULAR DISEASE

### 5.1 PSCI—ischemic stroke

Ischemic stroke is a cerebrovascular disease characterized by the sudden loss of blood flow to a region of the brain, leading to neuronal injury and tissue infarction. The primary neuroimaging modalities employed in radiomic analysis of ischemic stroke include CT and MRI.^129^

CT imaging, particularly non‐contrast CT, is widely used in the acute setting due to its rapid accessibility and ability to exclude hemorrhagic stroke. On the other hand, MRI, including DTI and perfusion‐weighted imaging, offers higher sensitivity in detecting early ischemic changes and assessing tissue viability, which are valuable in radiomic feature extraction to predict clinical outcomes and response to therapy.

Laboratory tests commonly associated with ischemic stroke include assessments of coagulation profiles (e.g., D‐dimer levels), lipid panels, and inflammatory markers such as C‐reactive protein (CRP). These tests provide additional biological markers that may correlate with stroke severity and prognosis. In some predictive models, genetic factors such as mutations in clotting factor genes are also considered.

The integration of imaging and laboratory data in radiomic analyses enables the development of comprehensive models that can enhance risk stratification, guide therapeutic interventions, and improve prognostic predictions for ischemic stroke patients.

#### 5.1.1 sMRI and CT‐based approach

The presentation of PSCI is heterogeneous, affecting a range of cognitive domains, such as those responsible for memory, language, attention, executive function, and visuospatial abilities. To date, the pathology of PSCI is not fully understood, but neuroanatomical damage from stroke, regardless of hemorrhagic and ischemic condition, is thought to be a primary factor influencing poststroke cognitive outcomes.^130^ Therefore, lesion location, which has been linked to the different types of cognitive deficits observed, is commonly used as radiomic features investigating PSCI. Lesion‐symptom mapping (LSM) studies aim to use mathematical mapping to elucidate the relationship between the location of brain lesions and the resultant subtypes of cognitive deficits, therefore building prediction model for the development of PSCI.^130^

Several radiomic studies have incorporated this technique into the LSM analysis (Table 3). Zhao et al. utilized SVR‐LSM to analyze neuroimaging data from 410 ischemic stroke patients and established associations between radiomic features, such as lesion locations, and specific poststroke cognitive deficits.^3^ The researchers identified critical brain areas associated with general cognitive functions and specific regions linked to specific cognitive domains, such as those responsible for language, memory, executive function, attention, and visuospatial deficits. For example, they found that general cognitive functions were associated with lesions in areas such as the angular gyrus, caudate, left pallidum, and anterior corona radiata. Lesions in dorsal cortical regions were significantly associated with language deficits, while left hemispheric lesions correlated with memory and executive function.

**TABLE 3:** Lesion‐symptom mapping study and the corresponding brain areas for specific cognitive deficits.

Literature	Image modality	Sample size	Algorithm used	Memory	Verbel	Attention	Executive function	Visual spatial
^131^	MRI	1075	Support vector regression + canonical correlation analysis	Left cingulate gyrus Left parietal Operculum cortex Left insular Left caudate Left amygdala	Left thalamus Left uncinate fasciculus			
^132^	MRI + CT	203	Support vector regression					Middle temporal gyrus Inferior temporal gyrus Superior temporal gyrus Pallidum Precentral gyrus
^133^	MRI	1080	Non‐negative matrix factorization + multi‐level Bayesian model	Left hippocampal and occipital regions	Left putamen Left globus pallus	Supramarginal and angular gyrus	Post‐central gyrus Lateral occipital and opercular cortices of the left hemisphere	Right hippocampal and occipital regions
^134^	MRI	147	Support vector regression	Left putamen Left hippocampus Optic radiation Para hippocampal gyrus	Left corticospinal tract putamen Precentral gyrus Left precentral gyrus Left corticospinal tract			
^135^	MRI	132	Ridge regression	Left basal ganglia Left thalamus Corpus callosum Right inferior frontal cortex	Left frontal, insula, temporal, parietal, lobe Bilateral basal ganglia Left thalamus Posterolateral cerebellum	Bilateral basal ganglia Bilateral thalamus Frontal eye field occipital cortex		Left occipital cortex Left frontal eye field Internal capsule and cingulate bundle splenium
^136^	MRI	145	Support vector regression	Middle frontal gyrus Posterior corona radiata Fornix Left insular Left pallidum	Anterior corona radiata Inferior fronto‐occipital fasciculus Left superior corona radiata Left parahippocampal gyrus	Anterior limb of internal capsule Inferior frontal gyrus (opercular) Superior frontal gyrus Fornix	Genu of corpus callosum Posterior limb of internal capsule Posterior thalamic radiation	Left tapetum Left putamen

Abbreviations: CT, computed tomography; MRI, magnetic resonance imaging.

Studies by Bonkhoff et al. and Arbula et al. extended these results by investigating hemispheric distinctions in lesion topographies and their associated cognitive deficits in acute ischemic stroke patients using similar SVM methods.^133^, ^137^ Their work illuminated the specific roles of the left and right hemispheres in language, memory, and visuospatial functioning. For instance, damage to the left hemisphere was particularly predictive of language and memory assessment outcomes, while damage to the right hemisphere was predictive of visuospatial functioning.

Other studies utilizing different ML techniques, such as deep learning with CNNs,^138^, ^139^ have also provided corroborative insights, with several neuroanatomical structures continuously being discovered to be related to specific cognitive deficit subtypes; these insights have reinforced the comprehensive and nuanced understanding of the relationship between lesion patterns and cognitive outcomes and might be beneficial for revealing the cause of PSCI from a neuroanatomical perspective.

Other than investigating the correlation between lesion location and cognitive impairment subtype, MRI‐based radiomics can also build prediction models for the development of PSCI. Jeong et al. applies deep feed‐forward networks analyzing MRI images for poststroke aphasia prediction in 176 ischemic stroke patients.^140^ Their model achieves remarkable accuracy in very severe (92%) and mild patient (70%), suggesting feasibility for estimating the severity of aphasia in the early stage of stroke, therefore allows early intervention in clinical treatment. Further research utilizes radiomic features from functional MRI connectivity to predict cognitive outcome.^131^ Their model successfully predicts the long‐term functional outcome in four different cognitive domines, including memory, attention, visuospatial functions, and language individually with promising accuracy, offering a more comprehensive predictive model compared to those only predict cognitive deficits in general. Although there is currently no consensus on the optimal ML method for PSCI prediction based on neuroimaging, it is clear that the accuracy of ML‐based radiomic models surpasses that of most traditional imaging analysis techniques and models. These studies further highlight the efficacy of ML as a potent tool for clinical use which allows early intervention.

After predicting the development of the disease, a sMRI‐based predictive model can also be used for PSCI rehabilitation outcome prediction. Dacosta‐Aguayo et al. employed a naive Bayesian tree classifier to predict the cognitive recovery of 27 ischemic stroke patients at 3 months post‐assessment using structural MRI connectome data.^141^ Their model, with 85.18% accuracy, underscored the significance of the structural connectivity between the left superior parietal gyrus and the left angular gyrus in recovery quality, thereby shedding light on the neural underpinnings of functional rehabilitation. Furthermore, Lai et al. focused on the recovery of specific cognitive domains affected by ischemic stroke, such as domains involved in aphasia.^142^ By combining sMRI and rs‐fMRI data with cognitive performance metrics, the researchers predicted language treatment outcomes using SVM and random forest classifiers. Their model accurately predicted patient responses to language treatment strategies with 80% accuracy, allowing for the selection of the most effective initial treatment strategy. The implications of this research are substantial. For example, by pinpointing crucial neural structures that affect rehabilitation, clinicians can target these areas to foster synaptic activity during treatment or protect them during surgeries required for stroke management. Prediction model outcome can also be used to guide the selection of therapies best suited to a patient's unique clinical profile, thus allowing treatments to be personalized for optimal efficacy.

#### 5.1.2 DTI‐based radiomic approach

While LSM studies provide detailed insights into the localization of specific brain areas and their associated cognitive deficits, contemporary neuroscience increasingly focuses on brain connectivity and networks.^143^ This perspective acknowledges that multiple brain regions are interconnected and cooperate within complex networks to facilitate cognitive processes. Solely focusing on isolated lesion areas while ignoring connections from a whole‐brain perspective may limit our understanding of the basic mechanisms of PSCI.^144^ Consequently, it is arguable that PSCI may arise because of disruptions in these neural networks due to stroke‐induced tissue damage, such as damage to white matter tracts in the context of PSCI, rather than being solely attributable to isolated lesions. The compensation mechanism in the brain would still be able to find a path enabling the communication between different regions, but in a much slower efficiency. Some research therefore hypothesized that this reduction in global efficiency would contribute to the general cognitive impairment in poststroke population.^144^ Nevertheless, the exact neural circuits contributing to the significant reduction in cognitive processing speed is yet to be discovered, and whether the lesion to different white matter tracts will result in different impairment subtype is unknown. Thus, understanding the broader impact of stroke on brain network integrity and function is imperative. ML offers sophisticated tools for analyzing changes in brain connectivity patterns poststroke through neuroimaging, potentially revealing insights into the network‐level correlations with underlying PSCI.

Several radiomic studies have incorporated ML into the analysis of brain connectivity through neuroimaging. The objective of these studies was to identify critical neuropathways contributing to the development of PSCI. Wang et al. utilized a sparse logistic regression classifier to analyze diffusion tensor imaging and arterial spin labeling data from 74 patients.^145^ The researchers discovered that the lateral capsular pathway of the cholinergic tracts and perfusion features predominantly located in the frontal‒subcortical‒limbic areas significantly contribute to patients' poststroke cognitive performance. Haller et al. conducted tract‐based spatial statistics analysis based on a SVM to evaluate the diffusive tensor imaging of 66 people with MCI (including ischemic stroke patients), aiming to discriminate different subtypes of cognitive impairment by white matter tract integrity.^146^ Their group‐level analysis suggested that people with PSCI have decreased functional anisotropy (FA) in the bilateral, right‐dominant network and a more pronounced reduction in FA in the right inferior frontal‒occipital fasciculus and inferior longitudinal fasciculus than controls.

Peng et al. used SVM to identify the unique characteristics of brain network topological connectivity in post‐hemorrhagic and ischemic stroke patients via functional near‐infrared spectroscopy covering the dorsolateral prefrontal cortex.^147^ The researchers discovered that functional connectivity strength, global efficiency, small‐world parameters, and nodal efficiency in the measured area were significantly lower in PSCI patients during cognitive tasks than in controls. These identified brain network characteristics could serve as integrated systems that collaboratively contribute to cognitive deficits in general. Additional studies have attempted to apply ML to more accessible neuroimaging techniques, such as electroencephalography.^148^, ^149^ These studies also achieved promising results in detecting abnormal brain connectivity and information processing speed in PSCI patients, identifying potential biomarkers for disrupted cognitive processes.

#### 5.1.3 Hybrid multimodel approach

Although models based solely on neuroimaging have yielded impressive results, researchers have recognized the importance of incorporating non‐imaging factors, such as PSCI risk factors, demographic features, and clinical characteristics, in predictive modeling.^150^ Consequently, another approach to predict stroke patients' cognitive outcomes is through a hybrid multimodel that accounts for a variety of information beyond neuroimaging alone.

In the study by Lee et al. acute ischemic stroke patients' neuroimaging profiles were merged with vascular risk factors such as arterial hypertension, along with information on dyslipidemia, diabetes mellitus, and potential cardiac embolism sources.^149^ The authors also included laboratory data, such as initial random glucose levels, white blood cell count, total cholesterol, and lipoprotein levels, as predictors of PSCI. By employing artificial neural networks and extreme gradient boosting algorithms, the researchers expanded the significant features from purely neuroimaging‐based methods to include stroke severity and history. Their model achieved notable accuracy, with values of 0.80 for the extreme gradient boost algorithm and 0.74 for the artificial neural network, demonstrating the effectiveness of utilizing multidimensional clinical data to predict PSCI.

Another study by Ji et al. provides a most comprehensive model to date. A total of 38 potential features, including neuroimaging data, were assessed for 397 ischemic stroke patients at the time of admission.^151^ Using a novel Gaussian naive Bayes classifier, the researchers pinpointed key predictive features, including CRP levels, homocysteine levels, and white matter degeneration. The accuracy of their model was validated in an external cohort, which was independent of the training data, enhancing its robustness and reliability. In the external validation set, their model yielded an accuracy of 0.86, with a sensitivity of 0.82 and specificity of 0.93, outperforming other classifiers and single‐dimensional neuroimaging models.

Furthermore, a hybrid multimodel for predicting poststroke rehabilitation outcomes has been used in some studies. In the study by Fast et al. a hybrid‐multimodal model combining 43 baseline clinical features, including neuroimaging data, was used to predict cognitive function recovery after a first‐ever ischemic stroke.^152^ The researchers used a SVM and identified crucial predictors for rehabilitation outcomes, such as the National Institutes of Health Stroke Scale score, smoking status, infarct pattern, and stroke origin. The model's predictions for recovery at discharge and at the 1‐year follow‐up were promisingly accurate, with an AUC of 0.7.

### 5.2 PSCI—hemorrhagic stroke

Hemorrhagic stroke is a type of cerebrovascular disease characterized by the rupture of blood vessels in the brain, leading to bleeding within the brain tissue (intracerebral hemorrhage) or the surrounding spaces (subarachnoid hemorrhage). Similar to the cognitive impairment in ischemic stroke, the primary imaging modality used in radiomics for hemorrhagic stroke is also MRI and CT based. Those image provides superior characterization of hemorrhagic lesions, detect microbleeds, and assess the extent of hematoma, which are crucial for radiomic feature extraction and predicting clinical outcomes.

Common laboratory tests in hemorrhagic stroke include coagulation profiles (e.g., prothrombin time, international normalized ratio), platelet counts, and inflammatory markers, such as interleukin‐6, which help evaluate the underlying pathophysiology and potential risk factors for hemorrhage. Genetic testing for conditions such as cerebral amyloid angiopathy or arteriovenous malformations may also be incorporated in predictive models. By combining neuroimaging data with laboratory test results, radiomic analyses can provide comprehensive insights into hemorrhage expansion, rebleeding risk, and overall prognosis, thereby aiding in personalized treatment planning and risk stratification for patients with hemorrhagic stroke

#### 5.2.1 sMRI and CT‐based radiomic approach

MRI and CT imaging have emerged as powerful tools for enhancing diagnostic and prognostic capabilities in cognitive impairment after hemorrhagic stroke. Al‐Mekhlafi et al. utilized SVM, KNN, decision trees, random forests, and multilayer perceptrons on MRI data, aiming for early detection of hemorrhagic stroke.^153^ All models showed promising prediction accuracy, with random forest algorithms achieving a precision of 98%.

Meng et al. further validated the efficacy of random forests in predicting hemorrhage transformation from ischemic stroke based on MRI images.^154^ Their model achieved an accuracy of 91%, suggesting the utility of ML prediction models in clinical settings. Importantly, their work indicates that the contralateral region of the lesion area plays a significant role in predicting hemorrhage transformation, a finding not previously reported. This suggests that even areas without lesions in normal regions of interest provide characteristic information for prediction, encouraging further research to take a holistic view when determining radiomic features.

Extending from development and transformation prediction, Hall et al. applied decision trees and random forests to CT and MRI data to identify modifiable predictors for patients' cognitive outcomes.^155^ Their analysis showed 85% accuracy and suggested that a hematoma volume less than 44.5 mL at admission is a positive predictor for better patient outcomes.

These studies collectively demonstrate that ML can capture nuanced details in neuroimages critical for predicting disease development and outcomes. The high accuracy demonstrated the potential application of ML models specifically in hemorrhagic stroke patients.

#### 5.2.2 Hybrid multimodel approach

Similar to ischemic stroke, a comprehensive multimodel combining both imaging and clinical data would provide more predictive value and accuracy towards the development of PSCI. However, there is currently a lack of research using multimodels to predict PSCI.

The influential work from Gu et al. incorporates MRI with clinical features including red blood cell (RBC) indices, neuropsychological tests, and functional tests to identify hemorrhagic stroke populations at risk of PSCI based on multivariate logistic regression.^156^ Their model achieves an accuracy of 91.4%. They also compare the performance with traditional univariate analysis.

Their results suggest that the ML approach significantly surpasses the traditional method, highlighting ML's ability to handle high‐dimensional data. They also identified that RBC indices are independent and important predictors of PSCI. A nomogram incorporating RBC indices can be used as a reasonable and reliable graphic tool to help clinicians identify patients at high risk of cognitive impairment and adjust individualized therapy.

### 5.3 Cerebral small vessel disease

CSVD is a common neurological condition characterized by damage to the small blood vessels in the brain, which often leads to cognitive impairment and an increased risk of stroke and dementia. This progression primarily affects the brain's white matter and deep gray matter, causing various forms of brain injury that are detectable through neuroimaging. Imaging modalities including sMRI and PET are commonly used due to their ability to provide detailed images of brain structure and function, highlighting abnormalities in tissue integrity and perfusion that are indicative of small vessel disease.

Clinical features other than neuroimaging are less investigated in ML‐based radiomics for cognitive impairment specific for CSVD. Some traditional research suggests that genetic predispositions, such as certain single nucleotide polymorphisms, and biomarkers such as homocysteine levels, inflammation markers (e.g., CRP), and neurofilament light chain can indicate the presence or risk of CSVD. However, there is currently a lack of multimodels that combine those features with neuroimaging to form a comprehensive predictive model.

#### MRI and PET‐based radiomic approach

MRI can reveal critical features for CSVD including white matter hyperintensities, perivascular spaces, cerebral blood flow, and perfusion. In PET imaging, amyloid and tau deposition can be identified. While more specific to AD, amyloid deposition can also be present in CSVD, especially in mixed pathologies. Several studies have incorporated these modalities in ML‐based radiomics to predict cognitive function for CSVD.

Several studies first attempt to identify the important features that have predictive value to the development of cognitive impairment in CSVD. Phuah et al. combined MRI and PET data to investigate the features that influence cognitive impairment.^157^ They applied a deep learning approach and identified that white matter hyperintensities spatial signatures may serve as etiology‐specific imaging markers for cognitive decline. They further suggest that the development of white matter hyperintensities burden across the supratentorial brain is not homogeneous, and spatial specificity of white matter hyperintensities reflects differential regional white matter vulnerability to injury from different disease pathologies. Shi et al. applied a logistic regression model based on MRI aiming to investigate the influence of cerebral blood flow, perivascular spaces, cerebral vessel pulsatility, and white matter hyperintensities on cognitive decline.^136^ Their results demonstrate that cerebral blood flow was not associated with CSVD features but support a close association of CSVD features with increased intracranial pulsatility rather than with low global cerebral blood flow, thus providing potential targets for mechanistic research, treatment, and prevention of CSVD.

Following this, the identified features can be used for classification of disease from health condition. For example, Wang et al. further investigated the contribution of white matter diffusion and cortical perfusion pathology to CSVD through sparse logistic regression classification. Their model achieved a classification accuracy of 72.57% in distinguishing cognitive impairment from healthy controls.^145^ They identified critical diffusion features largely spanning the capsular lateral pathway of the cholinergic tracts, and perfusion features mainly distributed in the frontal‒subcortical‒limbic areas. This mirrors the findings regarding cholinergic deficits in AD patients. Nevertheless, their findings suggest that disruption of white matter integrity might play a critical role in the progression of cognitive impairment in patients with CSVD.

### 5.4 Moyamoya disease

MMD is a chronic, progressive cerebrovascular disorder characterized by stenosis or occlusion of the terminal portions of the internal carotid arteries, leading to the development of a network of fragile collateral vessels known as moyamoya vessels. This pathological vasculature results in compromised cerebral perfusion and increased risk of ischemic and hemorrhagic events, contributing to significant morbidity and cognitive impairment.

Commonly employed radiomics modalities in MMD research include sMRI, fMRI, and PET. sMRI is instrumental in assessing the extent of cerebral atrophy, microstructural changes, and the presence of silent infarcts, which are critical in understanding the impact of MMD on cognitive functions. fMRI, on the other hand, provides insights into altered brain connectivity and functional reorganization, which are particularly relevant for assessing cognitive impairment associated with MMD. PET imaging, especially with tracers such as 18F‐FDG, offers a metabolic perspective, allowing for the evaluation of regional cerebral glucose metabolism, which is often disrupted in MMD patients and correlates with cognitive dysfunction.

In addition to imaging features, non‐imaging data such as clinical variables, CSF biomarkers, and genetic profiles play a crucial role in the comprehensive assessment of MMD. Clinical variables including patient demographics, neurological assessments, and cognitive test scores provide valuable context that complements imaging data.

#### 5.4.1 sMRI‐based radiomic approach

MRI could reveal several key features when investigating cognitive impairment in MMD. One common aim for this approach is to identify the critical radiomic features contributing to the development of cognitive impairment under MMD. For example, Tompkins et al. applied SVM and decision tree models to predict cognitive impairment based on cortical thickness abnormalities.^158^ They identified that the thickness of the rostral anterior cingulate, inferior part of the precentral sulcus, cuneus, caudal anterior cingulate, and inferior segment of the circular sulcus of the insula play key roles in cognitive decline.

Following the identification of those critical regions, studies aim to build classification models to distinguish MMD from normal control. For example, Akiyama et al. applied CNNs to analyze sMRI images, aiming to classify patients with MMD from those with atherosclerotic disease or normal controls.^159^ Their model achieved accuracies of 92.8%, 84.8%, and 87.8%, respectively, showing great potential for clinical use. Shen et al. (2022)^160^ used logistic regression to associate hypoperfusion with cognitive function in MMD patients without stroke.^157^ The strength of their work lies in the sophisticated participant recruitment, where the confounding influence of stroke was controlled to better investigate MMD's unique association with cognition. Their results suggest that cognitive function decline is significantly correlated with cerebral blood flow and hypoperfusion in the left centrum semiovale and temporal lobes.

#### 5.4.2 fMRI‐based radiomic approach

Several studies have utilized fMRI‐based radiomics to investigate important functional networks that contribute to cognitive decline in MMD. Gao et al. predicted changes in processing speed based on rs‐fMRI using connectome‐based predictive modeling.^161^ Their model showed that functional connectivity significantly predicts postoperative changes in processing speed at 1 and 6 months after surgery. Additionally, their study identified cerebrocerebellar and cortico‐subcortical connectivities that were consistently associated with processing speed. The brain regions identified from their predictive models are not only consistent with previous studies but also extend previous findings by revealing potential roles in postoperative neurocognitive functions in patients with MMD.

Chen et al. further integrated rs‐fMRI and DTI using a dual‐modal graph convolutional network module.^162^ Their model achieved an accuracy of 80% in identifying cognitively impaired patients with MMD. Their models also highlighted salient brain regions related to cognitive decline, including the basal ganglia, olfactory cortex, and hippocampus‐related regions. Lei et al. applied sparse representation‐based classification to recognize global cognitive impairment in MMD through high‐order dynamic functional connectivity networks.^163^ Their model reached 91.02% accuracy in identifying cognitive impairment in MMD compared to healthy controls, further suggesting that integrating time‐varying properties in dynamic functional connectivity networks from different orders has great clinical significance.

#### 5.4.3 PET‐based radiomic approach

Some studies have suggested that cognitive impairment in MMD follows a unique glucose metabolic pattern. Weng et al. applied a sparse representation‐based classifier to identify people with cognitive impairment among MMD patients based on 18F‐FDG‐PET imaging.^164^ Their model achieved an accuracy of 82.4% and revealed that hypometabolic lesions in the left hemisphere played a more important role in cognitive decline in patients with MMD. This work provides important clinical insight, demonstrating that PET, while not a primary imaging tool used in MMD, can also provide significant diagnostic value in assessing the development of cognitive impairment in MMD.

#### 5.4.4 Hybrid multimodel approach

Currently, there are few studies that utilize hybrid ML‐based multimodels to predict cognitive function in MMD patients. Limited evidence comes from Wang et al., who combined rs‐fMRI with cognitive and emotional assessments, aiming to classify patients with MMD using SVM model.^86^ The model achieved an accuracy of 67.5%. Despite the accuracy not being particularly promising, they identified the critical role of the angular cortex in cognitive impairment of MMD. This finding suggests that the angular cortex should be protected during surgical interventions.

## 6 CHALLENGES AND FUTURE DIRECTIONS IN MACHINE LEARNING‐BASED RADIOMICS FOR NEURODEGENERATIVE AND CEREBROVASCULAR DISEASES

### 6.1 Data‐related challenges: the foundation of reliable radiomics

#### 6.1.1 Sample size dilemma: balancing statistical power and practical constraints

ML has been extensively applied in PSCI imaging research, yet significant challenges and limitations persist. A primary issue is the lack of reproducibility for this technique. The discrepancy between studies may stem from various factors. Sample size is crucial for building accurate ML models. Unlike traditional methods and statistics, determining an efficient sample size for ML algorithms to achieve optimal power is highly complex.^165^ Moreover, there is no universal power analysis equation, and the ideal sample size can significantly differ based on the parameters of different ML algorithms.^165^ Sample size estimations typically involve simulations and comparisons, which are not entirely objective. The results may vary depending on the specific data analyzed. Another approach is to replicate sample sizes from similar studies, but this method also lacks objectivity, as it may involve the bias of literature selection. Furthermore, the difficulty in acquiring imaging data from stroke patients adds to this challenge, leading to significant variations in sample sizes across studies, potentially causing inconsistent findings. Hence, the research field lacks standard guidelines for estimating sample size; thus, developing a universal power analysis tool specific for ML algorithms is critical for improving the reliability of related studies.

To better estimate sample size in radiomic studies, Balki et al. proposed both pre hoc and post hoc approaches. In the pre hoc approach, Haykin suggested that a valid generalization is feasible if the condition *m* = *O*[(*d* + *k*)/*ε*] is met, where *m* represents the number of images, *d* represents the weight, *k* represents the units, and *ε* represents the classification error. In practical terms, this translates to a requirement of 240 training samples as a rule of thumb, aligning with the number proposed by Widrow's rule of thumb.^166^ For post hoc approaches, a learning curve‐fitting method was suggested, utilizing an inverse power law function to model the relationship between training data size and classification accuracy. This method aids in understanding how the performance of a ML algorithm improves with more training data, facilitating the identification of acceptable accuracy and the corresponding required sample size.

Other studies have proposed a general 1:10 ratio between the number of selected features and sample size^167^; however, this approach should be applied with caution, as the adequate sample size depends on various factors, including image quality, the algorithm applied, and the desired prediction outcomes. Relying on such a universal rule of thumb may lead to serious underestimation of the ideal sample size. Acquiring an adequate sample size is particularly challenging in real‐world research settings, especially in neuroimaging analysis. To mitigate this issue, the most common approach is to conduct dimensionality reduction via unsupervised learning before model training, effectively reducing redundant features by focusing on the most valuable components, and thus reducing the required sample size.^168^ Techniques such as principal component analysis or multidimensional scaling can play a critical role in this process. Additionally, transfer learning can be applied in scenarios where new data are used to fine‐tune or train previously pre‐trained models. Finally, multicenter data sharing across institutions is crucial to addressing sample size limitations, particularly in neuroimaging radiomic studies.

#### 6.1.2 Data quality and sharing: enhancing model robustness through collaboration

Model training performance in radiomics heavily depends on data quality, in addition to sufficient sample size.^169^ However, access to high‐quality neuroimaging is limited, and assembling comprehensive, high‐quality imaging data in a single study presents significant challenges. Differences in neuroimaging resolutions across studies can result in fundamentally different outcomes from ML model training processes.^3^ The variability in availability of advanced neuroimaging equipment among research teams further contributes to discrepancies in imaging quality, leading to inconsistencies in training ML models.

This challenge is particularly pronounced in less investigated diseases such as HD. Unlike AD and PD, which benefit from established databases such as Alzheimer's disease neuroimaging initiative (ADNI) and Parkinson's progression markers initiative (PPMI), HD lacks a similarly comprehensive resource. This deficiency makes it difficult to train robust ML models for HD. The scarcity of high‐quality imaging data from diverse populations limits the generalizability of developed models and can lead to overfitting.

To address these issues, establishing larger multicenter collaborations and data sharing initiatives is crucial. Such efforts, particularly when focused on underrepresented conditions such as HD, could significantly enhance the availability of relevant, high‐quality datasets. These collaborative approaches would not only increase the quantity of available data but also improve its diversity and quality, thereby enhancing the robustness and generalizability of radiomics models across various neurodegenerative and cerebrovascular conditions.

#### 6.1.3 Workflow standardization: toward reproducible radiomics research

Moreover, the workflow of radiomics introduces considerable variability to the field,^33^, ^170^, ^171^ which might lead to a low reproducibility rate and inconsistent results. This is primarily because radiomics studies can be conducted using either open‐source software platforms (such as MaZda or IBEX) or tools developed in‐house environment (such as in MATLAB). The differences in processing techniques, standardization methods, and the types and quantities of radiomic features extracted contribute significantly to the variability across studies.^33^ These variations make it challenging to reproduce results, compare findings among different studies, and integrate data for meta‐analysis. Currently, there is a lack of unified and standardized operational procedures for ML‐based radiomic analysis in neuroimaging. Future research could focus on developing best practice procedures and standardizing workflows to enhance consistency and reliability in the field.

### 6.2 Methodological hurdles in advanced radiomics

#### 6.2.1 Multimodal integration: harmonizing diverse imaging data for comprehensive analysis

The integration of multimodal imaging in radiomics offers a more comprehensive prediction model, but it also presents significant challenges, particularly in harmonizing data from different imaging techniques. This issue is evident across various neurodegenerative and cerebrovascular conditions, including AD and PSCI.

In AD research, studies often incorporate multiple imaging modalities such as PET and MRI. Each technique captures distinct aspects of AD pathology, and while combining these data types can enhance model accuracy, it also introduces complexity in data preprocessing and model tuning. Jiang and coworkers demonstrated that combining structural MRI with functional PET data can improve diagnostic performance.^172^ However, ensuring the robustness of these combined models and preventing overfitting due to increased dimensionality remains an ongoing concern.

Similarly, PSCI studies utilize various neuroimaging modalities, leading to diverse interpretations of brain activity. For instance, Wang et al.^145^ and Haller et al.^146^ analyzed neural connectivity using diffusion tensor imaging, while Hussain and Park^148^ and Lee et al.^149^ employed EEG. Peng et al.^147^ and Liao et al.^173^ explored regional topological organization using functional near‐infrared spectroscopy and rs‐fMRI, respectively. Each approach offers unique insights but also introduces discrepancies in data characteristics.

These differences in imaging modalities present significant challenges. EEG provides high temporal resolution but lacks fine spatial resolution for specific signal generation areas. Conversely, diffusion tensor imaging offers precise spatial resolution but cannot track real‐time cognitive processes. These fundamental differences in the nature of data fed into ML algorithms can lead to inconsistent results, particularly when considering the specific cognitive tasks employed in experiments.

To address these challenges, several approaches have been proposed. Developing standardized protocols for data acquisition and preprocessing can help mitigate variability between imaging modalities and enhance the reliability of combined models. Advances in data fusion techniques, such as multiview learning and transfer learning, show promise in effectively integrating diverse imaging data. Moreover, incorporating domain knowledge into model architecture design, such as embedding anatomical priors, can improve both the interpretability and effectiveness of multimodal approaches.

#### 6.2.2 Computational limitations: navigating the complexities of large‐scale neural network analysis

Despite advancements in computational technology, current ML radiomics can still struggle with very large datasets. This limitation is particularly evident in DTI‐based radiomics studies of PSCI. While DTI‐based radiomic studies provide detailed insights into how brain lesions affect global neural network connectivity and cognitive outcomes, there is no consensus yet on the brain network characteristics specific to PSCI. This inconsistency stems from several factors. Analyzing brain connectivity, especially in terms of topological patterns, requires substantial computational resources.^174^ Current ML models often struggle to accommodate vast amounts of data involving numerous nodes and pathways, leading to computational limitations. Due to these computational constraints, research often narrows its focus to local connections of specific brain areas known to significantly influence poststroke cognitive processes, such as the prefrontal cortex.^147^ This focused approach, while necessary given current limitations, may be subjective and cause broader neural connections to be overlooked. It yields only a partial view of the brain's connectivity network.

### 6.3 Disease‐specific challenges: tailoring radiomics to neurological complexities

#### 6.3.1 Heterogeneous disease presentations: adapting feature selection for diverse pathologies

The heterogeneity of disease presentation poses significant challenges for feature selection in radiomics, particularly in neurodegenerative conditions such as HD, PD, and LBD. Each of these conditions presents unique complexities that complicate the application of ML‐based approaches.

HD presents unique challenges due to its complex and progressive nature, involving widespread and variable brain atrophy affecting both gray and white matter structures. The subtle and diffuse neurodegenerative changes in HD are not as easily quantifiable as focal lesions seen in other conditions. This necessitates more sophisticated algorithms capable of capturing and interpreting complex, non‐linear patterns of degeneration. Deep learning techniques, which can automatically learn feature hierarchies from raw imaging data, may offer advantages in this context. However, they require large amounts of data and can be computationally intensive. Implementing transfer learning from models trained on other neurodegenerative diseases could provide a starting point, but these models must be carefully adapted and validated on HD‐specific data to ensure accuracy and relevance.

The feature selection process in PD is similarly complex. Unlike AD, which has more established structural and functional imaging changes, PD's neuroimaging markers are less pronounced and more variable across individuals, particularly in the early stages. This variability can lead to inconsistent model performance. Bian et al. observed that while ML can differentiate PD from other parkinsonian syndromes, accuracy levels often fluctuate due to this inherent variability in imaging features.^175^ To address this, the development of robust, disease‐specific radiomic features and the use of multimodal imaging (combining MRI, PET, and SPECT data) can help enhance the reliability of the models.

In LBD, the fluctuation in symptoms, both in terms of cognitive function and alertness, can significantly impact data quality and the timing of imaging studies. This variability can lead to inconsistencies in the radiomic features extracted, affecting the reliability of ML predictions. Addressing this requires not only the development of algorithms that can handle temporal variability but also the establishment of standardized imaging protocols that account for these fluctuations. Using repeated measures or longitudinal data might offer a way to capture the dynamic nature of LBD, allowing models to account for variability over time rather than relying on single‐time‐point assessments.

In conclusion, the heterogeneity of disease presentation across these neurodegenerative conditions underscores the need for tailored approaches in radiomics. Future research should focus on developing disease‐specific feature selection methods, leveraging advanced ML techniques, and incorporating longitudinal data to better capture the dynamic nature of these conditions. These efforts will be crucial in improving the accuracy and reliability of radiomic models in clinical applications.

#### 6.3.2 Inconsistent disease definitions: addressing variability in diagnostic criteria

The definition of certain disease can be heterogeneity. This could result in different sample characteristics across samples, which limit the comparability and integrability between studies. For example, the definition of PSCI, in both hemorrhagic and ischemic condition, is heterogeneous across studies (Table 4). Currently, a clear definition of PSCI is missing, which hinders a standardized and consistent subject recruitment in the field of research and may potentially lead to conflicting results and hinder meaningful outcomes.^176^, ^177^ There are several key factors in current research that often have discrepancies.

**TABLE 4:** Heterogeneity of poststroke cognitive impairment definition in the domine of diagnostic criteria and assessment time.

Literature	Stroke type	Diagnostic criteria	Time of cognitive assessment
^178^	Ischemic	MMSE < 25	3 months after stroke
^179^	Cerebral stroke	MMSE < 19 (illiteracy) and <22 (primary education)	3 months after discharge
^180^	Ischemic	MMSE < 24	3−6 month after stroke
^181^	Ischemic	MoCA < 24	Preceding 3−6 month
^182^	Ischemic + hemorrhagic	MoCA < 22	6−9 month after stroke
^183^	Ischemic	MoCA < 25	3 months after stroke
^176^	Hemorrhagic	MoCA < 26	12−14 days after stroke
^184^	Hemorrhagic	MoCA < 23	1 year with follow‐up

Abbreviations: MMSE, Mini‐Mental State Examination; MoCA, Montreal Cognitive Assessment.

The timing of cognitive assessment should be clearly defined and play a critical role in the accurate diagnosis of PSCI. Acute deficiencies in cognitive test scores are frequently observed immediately following a stroke, with improvements often noted upon retesting after several weeks. To account for this initial fluctuation and allow for stabilization of brain tissue damage, the final diagnosis of PSCI should be delayed until at least 6 months post‐event. This timeframe aligns with the chronic phase of stroke and is consistent with international classification systems, such as the American Psychiatric Association's Diagnostic and Statistical Manual, which recommends a delay in applying dementia diagnostic labels.

The severity of PSCI also lacks a clear cutoff to distinguish cognitive impairment and cognitive normal. For instance, the Montreal Cognitive Assessment (MoCA), a widely used cognitive screening tool, lacks universal cutoff scores to distinguish between PSCI. We believed identifying a rule‐of‐thumb that is special in stoke condition is critical, as it increases the comparability between studies. A recent meta‐analyses suggest that optimal MoCA cutoff scores for PSCI screening vary depending on the time elapsed since stroke onset: 26/25 within 1 month, 21/20 after 3−6 months, and 24/23 or 26/25 after 1 year.^185^ These findings underscore the importance of considering both the severity of cognitive impairment and the timing of assessment when diagnosing and managing PSCI.

#### 6.3.3 Research gaps in specific conditions: the case of hemorrhagic stroke

The number of studies in specific area is currently lacking, which warrants further research direction. The most pronounced lack is the imbalanced studies between ischemic stroke and hemorrhagic stroke. Current research has focused predominantly on ischemic stroke, with limited attention given to hemorrhagic stroke. Studies on the neuroimaging and poststroke cognitive conditions of hemorrhagic stroke patients are scarce. This gap in research may be partly due to the complexities in identifying lesion volumes and patterns in hemorrhagic stroke neuroimaging data, as the irregular shapes and patterns of hematomas, coupled with their mass effect, complicate analysis.^186^ Efforts to improve the identification of hemorrhagic stroke imaging features, such as by investigating patients in the chronic stroke stage when hematomas are fully absorbed or establishing criteria to exclude images with significant mass effects, have been initiated. Nonetheless, incorporating radiomic data from hemorrhagic strokes into ML analysis presents additional challenges and considerations. This issue is critical, as hemorrhagic strokes often result in more severe consequences than ischemic strokes, including higher mortality rates and more severe cognitive aftermath.^187^ Furthermore, ischemic and hemorrhagic strokes differ fundamentally in their pathologies and brain tissue damage mechanisms.^188^ Consequently, the findings from ischemic stroke radiomics cannot be seamlessly generalized to hemorrhagic stroke patients. The current understanding of the mechanisms and prognosis of hemorrhagic stroke remains inadequate, which poses significant challenges for clinical treatment and management. Future studies should specifically use hemorrhagic stroke patient data and analyze its unique lesion pattern and corresponding PSCI presentations.

Following this, we propose that when investigating PSCI using ML‐based radiomics, samples should not be mixed with both hemorrhagic and ischemic stroke patients. Betrouni et al. analyzed the texture features derived from T1‐weighted MR images of 327 PSCI patients (both hemorrhagic and ischemic) to train a random forest prediction model.^172^ This model achieved approximately 77% accuracy, suggesting that texture features from routine clinical MR images are reliable early indicators of PSCI. Chauhan et al. employed a hybrid model comprising CNNs, support vector regression, and ridge regression to predict the severity of language disorders directly from MR images of lesions in ischemic and hemorrhagic stroke patients.^138^ The model performance surpassed that of other methods, and tests confirmed its potential for predicting long‐term language deficits. The use of mixed samples in those studies was somewhat questionable despite its advantage in increasing sample size. When applying radiomics in clinical prognosis prediction, multiple imaging features were considered including texture, shapes, and intensities in comparison to neuro‐correlates research where lesion location was primarily focused. Those imaging features vary largely in different stroke types. When using a mixed sample, it increases the heterogeneity of the data, making it difficult for the ML algorithm to learn relevant features effectively. In addition, treatments and rehabilitation strategies might differ between hemorrhagic and ischemic stroke patients. Predictive models that do not account for these differences may not be clinically useful or could lead to inappropriate recommendations for patient care.

### 6.4 Balancing model complexity and clinical utility

The interpretability of the current ML models also draws concerns. For example, a common practice in the studies mentioned involves selecting models based on multiple comparisons to determine prediction accuracy across various ML algorithms, often favoring the most accurate model.^138^ However, this approach leads to a “black box” scenario, where the reasons behind the superior performance of certain models remain unclear. This lack of transparency extends to the medical interpretation of predictive outcomes and the identification of key radiomic features deemed significant by models.^152^, ^189^ For instance, while multiple studies have identified factors such as the neural inflammatory response index, including CRP, as critical predictors for PSCI diagnosis and rehabilitation,^151^, ^190^ the underlying mechanisms linking inflammation to PSCI have not yet been explored. This gap in understanding results in a lack of concrete evidence to guide model selection for specific clinical scenarios, such as varying stroke types, locations, and cognitive deficits. Additionally, the absence of clear explanations for secondary results from ML‐based modeling leads to a somewhat blind approach when applying ML‐based radiomics. Such limitations raise concerns about the reliability and robustness of using these models in practical clinical situations.

In addition, it is arguable that the accuracy of a model should be the only indicator of its performance. There is an ongoing trend in the research field to continually add more predictors to create hybrid multimodels. However, this approach is indeed paradoxical; though increasing the number of predictors can enhance a model's comprehensiveness and enable the algorithm to capture variables overlooked by traditional analysis, this method does not always translate into a better model. The accuracy and the statistical robustness of a model tend to increase as more predictors are included, but this can be accompanied by risks of overfitting and noise, which undermines the model's generalizability.^191^ In clinical settings, the utility of a model extends beyond mere accuracy; its predictions must be interpretable and meet the needs of healthcare professionals. Overly complex models with excessive predictors may not be practically useful, despite their high accuracy; therefore, they fail to provide insights into the underlying mechanisms of diseases such as PSCI. Current research seems to prioritize achieving the highest accuracy, occasionally overlooking the importance of understanding the underlying mechanisms, which is vital for advancing clinical treatments. This trend deviates from the original goal of using ML to evaluate PSCI data, which is to assist in diagnosis and prognosis.

### 6.5 Bridging research and practice: the path to clinical implementation

The journey toward widespread clinical use of ML‐based radiomics in PSCI is still challenging. Despite ML not being in its infancy, there are very few clinical applications. The integration of ML techniques into clinical workflows faces considerable hurdles due to the lack of established guidelines. Implementing these techniques in the standard operational procedure may require substantial changes, which may include the training of healthcare staff, modifications to electronic health record systems, and alterations to diagnostic and treatment protocols. This suggests that clinicians must not only possess advanced clinical skills but also acquire some fundamental computational knowledge. Such a combination could effectively harness the capabilities of modern computational techniques in real‐world clinical settings. In addition to the necessary improvements in medical staff and systems, advancements in ML algorithms are needed to address the “black box” issue and make ML‐based radiomics widely applicable. The development of more clear AI techniques with enhanced interpretability is crucial for understanding the mechanisms of disease and improving clinical applicability.^192^ This may necessitate cross‐disciplinary collaboration involving experts in data science, neuroscience, and clinical medicine, fostering a holistic approach to tackling complex challenges in PSCI research. Such collaboration would not only demystify the inner workings of ML models but also integrate diverse perspectives and expertise, leading to more robust, interpretable, and clinically relevant solutions.

Despite its limitations, ML‐based radiomics holds significant promise for applications in PSCI. Recent studies in this field have demonstrated a growing trend toward using larger datasets, which aim to enhance the generalizability and accuracy of predictive models Song et al.^193^ Collaborative research across institutions is particularly valuable, providing insights into the real‐world utility of these models in clinical settings. Efforts have also been made to standardize protocols in brain imaging radiomic studies, with guidelines such as those proposed by Carré et al. being increasingly adopted by researchers.^194^

Moreover, there is a strong emphasis within the scientific community on advancing ML and AI techniques. Each year, new developments and improvements are proposed to address current limitations.^195^, ^196^ Keeping abreast of the latest algorithms is crucial for ensuring the novelty and interpretability of models in radiomic research. Overall, the potential of ML techniques in radiomics is vast. With ongoing efforts to address current challenges, these techniques have the potential to be feasibly and effectively applied in clinical settings.

## 7 CONCLUSION

The integration of ML techniques with radiomics has opened new avenues for understanding and managing cognitive impairments associated with neurodegenerative and cerebrovascular diseases. This review highlighted the potential of ML to enhance early diagnosis, improve disease classification, and predict progression in conditions such as AD, PD, LBD, HD, and PSCI.

ML models, particularly those based on high‐dimensional neuroimaging data, have demonstrated superior performance compared to traditional approaches, offering more nuanced and accurate predictions. For neurodegenerative diseases, these models have been instrumental in detecting specific biomarkers, such as amyloid plaques in AD and DAT deficiencies in PD. In cerebrovascular conditions, ML aids in mapping the relationships between lesion locations and cognitive outcomes, providing valuable insights into the structural and functional disruptions underlying cognitive deficits.

However, the application of ML in this domain faces several challenges, including data heterogeneity, limited sample sizes, and the complexity of integrating multimodal data sources. Moreover, while promising, these approaches are not without limitations; issues such as overfitting, generalizability across diverse populations, and the need for more standardized data acquisition protocols remain critical barriers to clinical implementation. Additionally, the variability in radiomic features and disease presentations, particularly in conditions such as LBD, further complicates the predictive modeling efforts.

Future research should focus on addressing these limitations by expanding datasets through multicenter collaborations, developing more sophisticated algorithms capable of handling diverse data types, and standardizing imaging and diagnostic protocols. The use of advanced data fusion techniques and incorporating domain knowledge into model design could also enhance the interpretability and clinical relevance of ML models.

Overall, while challenges remain, the potential of ML‐based radiomics to revolutionize the diagnosis and management of cognitive impairments is immense, promising more personalized and effective approaches to care in the near future.

## AUTHOR CONTRIBUTIONS

*Writing the manuscript, literature search, reviewing the comment, and editing the manuscript based on comment*: M.‐G.S. *Literature search, formatting the paper, reference validation, and reference editing*: X.‐M.F., H.‐Y.Z., and L.H. *Supervise the writing, provides guidance on the topic, proofread the manuscript, editing the manuscript, and provides feedback and guidance on revising the manuscript*: D.‐F.F. All authors have read and approved the final manuscript.

## CONFLICT OF INTEREST STATEMENT

The authors declare they have no conflict of interest.

## ETHICS STATEMENT

Not applicable.

## Data Availability

Not applicable.
